# Evolution of Tandem Repeats Is Mirroring Post-polyploid Cladogenesis in *Heliophila* (Brassicaceae)

**DOI:** 10.3389/fpls.2020.607893

**Published:** 2021-01-12

**Authors:** Mert Dogan, Milan Pouch, Terezie Mandáková, Petra Hloušková, Xinyi Guo, Pieter Winter, Zuzana Chumová, Adriaan Van Niekerk, Klaus Mummenhoff, Ihsan A. Al-Shehbaz, Ladislav Mucina, Martin A. Lysak

**Affiliations:** ^1^CEITEC, Masaryk University, Brno, Czechia; ^2^NCBR, Faculty of Science, Masaryk University, Brno, Czechia; ^3^Department of Experimental Biology, Faculty of Science, Masaryk University, Brno, Czechia; ^4^South African National Biodiversity Institute (SANBI), Kirstenbosch, Cape Town, South Africa; ^5^Institute of Botany, Czech Academy of Sciences, Prùhonice, Czechia; ^6^Department of Geography & Environmental Studies, Stellenbosch University, Stellenbosch, South Africa; ^7^Department of Biology, Botany, Osnabrück University, Osnabrück, Germany; ^8^Missouri Botanical Garden, St. Louis, MO, United States; ^9^Harry Butler Institute, Murdoch University, Perth, WA, Australia

**Keywords:** repetitive DNA, repeatome, whole-genome duplication (WGD), rDNA ITS, plastome phylogeny, Cruciferae, Cape flora, South Africa

## Abstract

The unigeneric tribe Heliophileae encompassing more than 100 *Heliophila* species is morphologically the most diverse Brassicaceae lineage. The tribe is endemic to southern Africa, confined chiefly to the southwestern South Africa, home of two biodiversity hotspots (Cape Floristic Region and Succulent Karoo). The monospecific *Chamira* (*C. circaeoides*), the only crucifer species with persistent cotyledons, is traditionally retrieved as the closest relative of Heliophileae. Our transcriptome analysis revealed a whole-genome duplication (WGD) ∼26.15–29.20 million years ago, presumably preceding the *Chamira*/*Heliophila* split. The WGD was then followed by genome-wide diploidization, species radiations, and cladogenesis in *Heliophila*. The expanded phylogeny based on nuclear ribosomal DNA internal transcribed spacer (ITS) uncovered four major infrageneric clades (A–D) in *Heliophila* and corroborated the sister relationship between *Chamira* and *Heliophila*. Herein, we analyzed how the diploidization process impacted the evolution of repetitive sequences through low-coverage whole-genome sequencing of 15 *Heliophila* species, representing the four clades, and *Chamira*. Despite the firmly established infrageneric cladogenesis and different ecological life histories (four perennials vs. 11 annual species), repeatome analysis showed overall comparable evolution of genome sizes (288–484 Mb) and repeat content (25.04–38.90%) across *Heliophila* species and clades. Among *Heliophila* species, long terminal repeat (LTR) retrotransposons were the predominant components of the analyzed genomes (11.51–22.42%), whereas tandem repeats had lower abundances (1.03–12.10%). In *Chamira*, the tandem repeat content (17.92%, 16 diverse tandem repeats) equals the abundance of LTR retrotransposons (16.69%). Among the 108 tandem repeats identified in *Heliophila*, only 16 repeats were found to be shared among two or more species; no tandem repeats were shared by *Chamira* and *Heliophila* genomes. Six “relic” tandem repeats were shared between any two different *Heliophila* clades by a common descent. Four and six clade-specific repeats shared among clade A and C species, respectively, support the monophyly of these two clades. Three repeats shared by all clade A species corroborate the recent diversification of this clade revealed by plastome-based molecular dating. Phylogenetic analysis based on repeat sequence similarities separated the *Heliophila* species to three clades [A, C, and (B+D)], mirroring the post-polyploid cladogenesis in *Heliophila* inferred from rDNA ITS and plastome sequences.

## Introduction

Geographically and phylogenetically well-defined groups are ideal study objects to analyze the evolution of diverse genomic parameters during long periods of isolation that prevented gene flow with other species groups. Although Brassicaceae (mustard family, Cruciferae) occur on all continents, except for Antarctica, and several weedy and crop species have a worldwide distribution, some crucifer clades are restricted to (sub)continents or smaller geographic regions (Lysak and Koch, 2011; Al-Shehbaz, 2012). For instance, tribes of the CES clade (i.e., Cremolobeae, Eudemeae, and Schizopetaleae), as well as Halimolobeae, Physarieae, and all but one Thelypodieae species, are endemic to the New World, while Microlepidieae occur only in Australia and New Zealand. In Africa, the family has a reduced species and generic diversity, with the largest endemic clade confined to southern Africa (South Africa, Lesotho, eSwatini, and Namibia). The tribe Heliophileae includes some 104 species (compilation by I. A. Al-Shehbaz) concentrated chiefly in the winter-rainfall region of the southwestern South Africa, home to two global biodiversity hotspots – Cape Floristic Region and Succulent Karoo. *Heliophila* ranks among the largest crucifer genera, such as *Alyssum*, *Boechera*, *Cardamine*, *Draba*, *Erysimum*, *Lepidium*, and *Physaria* (Al-Shehbaz, 2012). The genus is often regarded as morphologically the most diverse Brassicaceae lineage (Mummenhoff et al., 2005). *Heliophila* varies from small ephemeral annual to perennial herbs (incl. one lianella), subshrubs, and tall shrubs (e.g., *Heliophila brachycarpa*). The species vary particularly in foliage (entire to variously dissected); petal length (1.2–30 mm) and color (white, pink, mauve, purple, blue, or yellow); number and presence vs. absence of petal and stamen appendages; presence vs. absence of paired glands at the bases of pedicels and/or leaves; ovule number (1–80); fruit length (2–120 mm long), shape (linear, lanceolate, oblong, ovate, elliptic, orbicular), constriction (moniliform or not), type (silique, silicle, samara, schizocarp), and flattening (terete, quadrangular, latiseptate, angustiseptate); gynophore length (obsolete to 12 mm long); style length (0.3–20 mm long) and shape (linear, filiform, conical, clavate, ovoid, globose); seed length (0.6–9 mm long), shape, and development of wing; and cotyledonary type (diplecolobal, spirolobal) (Marais, 1970; Mummenhoff et al., 2005; Mandáková et al., 2012; unpublished data).

Despite *Heliophila* species being a frequent and sometimes dominating element of some southern African plant communities, there is limited knowledge of the phylogeographic origin of the genus, interspecies relationships, and genome evolution of *Heliophila* species. Mummenhoff et al. (2005) published a pioneering study, laying foundations for follow-up phylogenomic analyses, demonstrating monophyly of the tribe Heliophileae with South Africa’s endemic *Chamira circaeoides* as the sister species to *Heliophila*, finding support for rapid diversification against a background of aridification in the Pliocene/Pleistocene, and showing massive parallel evolution of fruit characters traditionally used in the classification of Heliophileae. Further, ecological optimization analysis allowed preliminary insights into the ecogeographical evolution in Heliophileae.

The last phylogenetic study of c. 57 *Heliophila* species based on internal transcribed spacer (ITS) sequences suggested basal polytomy involving three clades, all sister to *Chamira* (Mandáková et al., 2012). The latter authors showed that two ITS clades are dominated by two chromosome numbers (2*n* = 20 and 2*n* = 22), whereas the third clade mainly contained shrubby species with chromosome numbers known only for two species at that time. Chromosome numbers in 27 analyzed *Heliophila* species ranged from 2*n* = 16 to 2*n* = c. 88, presumably due to polyploidy and dysploidal chromosomal rearrangements. Interestingly, comparative chromosome painting analyses, revealing the duplicated nature of *Heliophila* genomes, suggested the existence of an allohexaploid ancestor preceding the divergence of *Heliophila* lineages (Mandáková et al., 2012). This was supported by an analysis of synonymous substitution rates (*Ks*) of paralogous and orthologous genes in *Heliophila* cf. *longifolia* (Mandáková et al., 2017).

The high species diversity (>100 species) and extraordinary ecomorphological variability of *Heliophila* impacted by ancient and more recent whole-genome duplication (WGD) events and following post-polyploid diploidization (PPD), confined to one of the most remarkable biodiversity hotspots, make the genus an intricate but attractive phylogenomic model. In this study, based on the previous results and by including a broader spectrum of species, we aim at providing new insight to the WGD–PPD process, test the robustness of the inferred infrageneric relationships (Mummenhoff et al., 2005; Mandáková et al., 2012), and analyze the evolution of repetitive DNA sequences. Based on the updated ITS phylogeny, we selected 15 *Heliophila* species, representing the major infratribal ITS clades, for low-coverage whole-genome sequencing (lcWGS). Using lcWGS data, we reconstructed a dated whole-plastome phylogeny and characterized the most abundant repetitive sequences (repeatomes) of 15 *Heliophila* species and the sister *C. circaeoides*. We tested whether the ITS-based infrageneric clades are congruent with the plastome phylogeny and phylogenetic relationships inferred from repeat sequence similarities (Vitales et al., 2020). Further, we analyzed the repeat diversity and abundances in relation to the post-polyploid cladogenesis in *Heliophila*. The inclusion of *C. circaeoides*, the only crucifer species with persistent cotyledons, allowed us to get a first insight into its genome.

## Materials and Methods

### Plant Material

The list of all analyzed *Heliophila* and *Chamira* accessions, and outgroup species, is provided in Supplementary Table 1. Errors in the determination of species names for accessions used in previous phylogenetic analyses (Mummenhoff et al., 2005; Mandáková et al., 2012) were investigated and revisited where necessary. Selected 15 *Heliophila* species and *C. circaeoides* were used for detailed phylogenetic, repeatome, and cytogenetic analyses (Supplementary Table 2).

### Genome Size Estimation

Holoploid genome sizes were estimated by flow cytometry in species from which we had seeds and could grow plants in a greenhouse (Supplementary Table 2). One sepal (if available) or a fully developed intact leaf was prepared according to Doležel et al. (2007), and isolated nuclei were stained using propidium iodide + RNase IIA (both 50 μg/ml) solution, for 5 min at room temperature, and analyzed using a Partec CyFlow cytometer. A fluorescence intensity of 5,000 particles was recorded. *Solanum pseudocapsicum* (1C = 1.30 pg; Temsch et al., 2010) served as the primary reference standard. One individual of each species measured on three consecutive days was analyzed.

### Transcriptome Sequencing and Analyses of Whole-Genome Duplication

Total RNA was extracted from *H. lactea*, *H.* cf. *longifolia*, *H. seselifolia* subsp. *nigellifolia*, and *C. circaeoides* (Supplementary Table 3) using RNeasy Plant Mini Kit (Qiagen). Strand-specific library preparation (Illumina Truseq Stranded mRNA) and RNA-Seq (Illumina MiSeq, paired-end reads, 2 × 300 bp) were performed at the Oklahoma Medical Research Foundation (Oklahoma City, United States). Raw reads were corrected with Rcorrector v1.0.4 (Song and Florea, 2015) and trimmed with Trimmomatic v0.36 (Bolger et al., 2014) to remove low-quality reads and potential adapters. *De novo* assembly of transcriptomes was carried out with Trinity v2.5.1 (Haas et al., 2013) with default settings. Assembly summary statistics can be found in Supplementary Table 3. We excluded low-quality transcripts detected by Transrate v1.0.3 (Smith-Unna et al., 2016), removed chimeric transcripts, and clustered the remaining transcripts with Corset v1.07 (Davidson and Oshlack, 2014) after mapping RNA-Seq reads with Salmon v0.9.1 (Patro et al., 2017). Coding sequences (CDS) were predicted from the longest sequence of each cluster by TransDecoder v5.0.2 (Haas and Papanicolaou, 2016). Potentially redundant sequences (identity higher than 99%) were further removed with CD-HIT v4.7 (Fu et al., 2012). Gene completeness was then assessed by BUSCO v4.1.2 (Simão et al., 2015). For comparative purposes, we also included publicly available genome of *H.* aff. *coronopifolia* (Kiefer et al., 2019) into the downstream analyses.

To investigate the timing of speciation and potential WGD events in *Chamira* and *Heliophila*, we analyzed synonymous substitutions per synonymous site (*Ks*) for paralogous and orthologous gene pairs identified from within- and between-species comparisons, respectively, using the wgd pipeline (Zwaenepoel and Van de Peer, 2019). We also estimated the heterozygosity of coding genes by detecting SNPs with the GATK v4.0.1.0 pipeline (Poplin et al., 2017). Base Quality Score Recalibration built in GATK was used to detect systematic errors in accuracy of each base call during sequencing. The following filters were applied in GATK when detecting SNPS: QD < 2.0, FS > 60.0, MQ < 40.0, and SOR > 4.0. For each species, RNA-Seq reads were mapped to their respective CDS by Bowtie2-2.3.0 (Langmead and Salzberg, 2012). To allow a more direct comparison, we used OrthoFinder pipeline (Emms and Kelly, 2015) to identify 1-to-1 orthologs shared by *C. circaeoides* and *Heliophila* species. We excluded *H.* aff. *coronopifolia* (Kiefer et al., 2019) from the heterozygosity analysis because there were no RNA-Seq reads available.

### Nuclear Gene Phylogeny and Phylogenetic Reconciliation

For phylogenetic analyses, we complemented our CDS dataset (five species mentioned above) with 15 additional species of Brassicales which had public genomic data available (Kiefer et al., 2019; Supplementary Table 4), including *Tarenaya hassleriana* (Cleomaceae) as an outgroup. Following Yang and Smith (2014), we inferred sequence homology by all-against-all BLASTn search and filtered the output with a hit fraction of 0.3. We employed MCL v14-137 (Van Dongen and Abreu-Goodger, 2012), with parameters “-tf ‘gq(5)’ -I 1.4,” to obtain putative homologous gene clusters. The clusters with a minimum of 15 taxa were aligned using MAFFT v7.450 (Katoh and Standley, 2013) with the settings –genafpair and –maxiterate 1,000. The alignment columns with more than 90% missing data were removed using the Phyx software (Brown et al., 2017). We built a maximum-likelihood tree using a concatenated alignment of 37 single-copy genes with IQ-TREE v1.6.10 (Nguyen et al., 2014), with 1,000 rapid bootstrap replicates. ModelFinder (Kalyaanamoorthy et al., 2017) was used to identify the best fitted substitution model. We also built gene trees separately and inferred coalescent-based phylogeny with ASTRAL v5.7.3 (Zhang et al., 2018). For homologous gene groups with multiple copies in one species, we explicitly selected for those with higher copy number (> = 2) in *Chamira* + *Heliophila* species and single copy in the remaining species. To test for the mode of WGD, we converted the gene trees to multilabeled ones and performed phylogenetic reconciliation using GRAMPA v1.3 (Thomas et al., 2017). The ASTRAL topology was used as the input of species tree hypothesis.

### Ribosomal Internal Transcribed Spacer Phylogeny

The ITS1 and ITS2 regions were newly sequenced in 102 *Heliophila* accessions, and the obtained sequences (Supplementary Table 1; GenBank accession numbers MW216680–MW216783 for ITS1 and MW216784–MW216887 for ITS2) were combined with data published earlier (Mandáková et al., 2012). Methods for DNA extraction, PCR amplification, and ITS sequencing followed Mummenhoff et al. (2004). Multiple alignment of ITS sequences was generated using MAFFT v7.450 and then manually checked and trimmed. Bayesian inference from ITS alignment was performed using MrBayes XSEDE v3.2.7a (Ronquist et al., 2012) at CIPRES Science Gateway (Miller et al., 2010; Towns et al., 2014). Two independent Markov chain Monte Carlo (MCMC) analyses under the GTR+I+G model were run for 200 million generations, chains sampling every 5,000 generations, and burn-in 0.25. Convergence diagnostics for MCMC were conducted by Tracer v1.7.1 (Rambaut et al., 2018).

### Low-Coverage Whole-Genome Sequencing

NucleoSpin Plant II kit (Macherey-Nagel) was used to extract the genomic DNA from fresh or silica-dried leaves. DNA sequencing libraries were prepared and sequenced at the sequencing core facility of the Oklahoma Medical Research Foundation (Oklahoma City, United States). The Illumina MiSeq platform, generating 151-bp paired-end reads, was used for sequencing.

### Chloroplast Genome Assembly and Divergence Time Estimated Phylogeny

We assembled complete chloroplast (cp) genomes for 15 *Heliophila* species, *C. circaeoides*, and *Subularia aquatica* using NOVOPlasty v3.2 (Dierckxsens et al., 2016), using the *ndhF* gene of *Arabidopsis thaliana* (GenBank: NC_000932.1) as the seed (Supplementary Table 5). The cp genomes were annotated by plann v1.1.2 (Huang and Cronk, 2015) with *A. thaliana* as the reference genome, which was followed by manual curation using the Sequin software^1^.

We retrieved cp genomes of additional 42 Brassicaceae species from GenBank, representing all major Brassicaceae lineages, to investigate the maternal phylogeny of *Heliophila* within the whole family. A total of 103 genic and 102 intergenic regions were extracted from multiple-sequence alignment generated by MAFFT v7.450 with the L-INS-i mode. Gblocks v0.91b (Talavera and Castresana, 2007) was used to remove poorly aligned regions with a minimum block length of 2 bp. We subsequently concatenated the alignments and selected the best partitioning scheme with PartitionFinder v2.1.1 (Lanfear et al., 2016). A maximum-likelihood (ML) tree was reconstructed using IQ-TREE v1.6.10 with three *Aethionema* species as outgroup. Following Guo et al. (2017), we performed molecular dating with MCMCTREE (Rannala and Yang, 2007). The root age was set to 31.2 million years ago (Mya) according to Hohmann et al. (2015). The burn-in period was set to 2,000,000 cycles, and the MCMC run was sampled every 800 cycles until a total of 10,000 samples were collected. Diagnostics for MCMC were performed by Tracer v1.7.1.

### Preprocessing and Cluster Analysis of Repetitive DNA From Next-Generation Sequencing Data

Quality checks were performed with FastQC v0.11.7 (Andrews, 2010). Illumina adapter removal, quality filtering with 90% of bases equal to or above the quality cutoff value of 20, and trimming procedures were performed with Trimmomatic v0.36. All reads were quality filtered and trimmed down to 140 bp. Using Bowtie2-2.3.0 aligner software, organelle DNA that originated from chloroplast and mitochondria were filtered out prior to the analysis. Characterization and analysis of repetitive DNA were conducted using the graph-based clustering pipeline RepeatExplorer2 (Novák et al., 2013) as described by Novák et al. (2010, 2017).

Clustering was performed using 90% similarity over 55% of the read length as default settings. This analysis resulted in the construction of clusters that represent different repetitive DNA families. All sequences that built the clusters were in the form of contigs. Clusters with a genome proportion higher than 0.01% were annotated in detail. The maximum number of reads was used to perform detailed annotations in individual species to identify all repetitive sequences. Comparative clustering analysis was performed with concatenated next-generation sequencing (NGS) reads of 15 *Heliophila* and the sister species *C. circaeoides* (Supplementary Table 2). To avoid the coverage bias in the comparative repeatome analysis, preprocessed paired-end reads were randomly sampled in order to represent 10% of a genome (i.e., coverage = 0.1×) based on (1C) genome sizes (Novák et al., 2010). The same RepeatExplorer2 settings were used in the comparative analysis with individual clustering analysis.

Repetitive DNA cluster annotations were done by RepeatExplorer2 pipeline using DNA and protein similarity searches on clusters with known protein domains. Clusters which could not be classified by the pipeline were manually annotated using BLAST (Altschul et al., 1990) searches against the GenBank sequence and Censor (Kohany et al., 2006) databases. Clusters which were annotated as tandem repeats (directly or manually from the shape of the cluster graph) were further tested with Tandem Repeat Finder software (Benson, 1998) and similarity dot-plots with Dotter (Sonnhammer and Durbin, 1995). Tandem Repeat Analyzer (TAREAN, Novák et al., 2017) which is implemented in RepeatExplorer2 pipeline was used to reconstruct consensus monomers of the tandem repeats. All annotations were revised and corrected if necessary. Subsequently, all identified tandem repeats from all species were compared with each other using BLASTn searches to detect shared tandem repeats.

### Phylogeny Based on Repeatome Similarity

The novel phylogeny inference method using repeatome similarities as a source of phylogenetic marker was performed as introduced by Vitales et al. (2020). This method is based on the pairwise genetic distances between repeatomes of closely related species. By calculating the observed/expected number of edges (of similarity) between all species for each cluster from the output of RepeatExplorer2 comparative analysis, a similarity matrix is generated and transformed into distance matrices by calculating the inverse of the values (Vitales et al., 2020). Three datasets were created: 15 *Heliophila* species from all clades (A–D); 9 species from clades A, B, and D; and 9 species from clades B, C, and D. Subsequently, neighbor-joining trees were constructed for the clusters which included repeats that were present in all species out of the first 100 clusters using R (R Core Team, 2013) and *ape* package (Paradis and Schliep, 2019). The trees were then used to construct a consensus tree for each dataset with the SplitsTree4 v4.14.6 software (Huson and Bryant, 2006). Lastly, consensus tree including all *Heliophila* species was transformed to a dendrogram for a better representation.

Further, using RepeatExplorer2 comparative analysis, read abundance matrix, hierarchical cluster analysis was performed using *pheatmap* package (Kolde and Kolde, 2015) in R. The abundance matrix was transformed into a distance matrix by *pheatmap*. The clusters with genome proportion higher than 0.01% were used to construct the dendrogram relationship of 15 *Heliophila* and *C. circaeoides* species. *pheatmap* package in R was used to construct the heatmap.

### Chromosome Preparations

Young inflorescences were collected from plants in the field. Inflorescences were fixed in freshly prepared fixative (ethanol:acetic acid, 3:1) overnight, transferred into 70% ethanol, and stored at −20°C until used. Chromosome spreads from young fixed flower buds, containing immature anthers, were prepared according to the published protocol (Mandáková and Lysak, 2016a).

### DNA Probes

The list of all the designed probes and primers specific to repetitive elements is provided in Supplementary Table 6. Synthetic oligonucleotide probes were used for tandem repeats with shorter monomers (<500 bp). Target sequences (60 nt) with GC content 30–50% were selected from DNA alignments using Geneious v11.1.5 software package^2^ to minimize self-annealing and formation of hairpin structures. DNA probe preparation and labeling followed the published protocol (Mandáková and Lysak, 2016b). For satellites with longer monomers, PCR primers were designed to face outward from the monomer; therefore, PCR amplification was performed only between monomers tandemly arrayed. For retrotransposons, PCR primers were designed to the GAG domain which is generally the most variable domain among different retrotransposon families. PCR products were purified using NucleoSpin Gel and PCR Clean-up kit (Macherey-Nagel) and labeled by nick translation.

### Fluorescence *in situ* Hybridization, Microscopy, and Image Processing

Twenty microliters of the hybridization mix containing 100 ng of the labeled probe dissolved in 50% formamide and 10% dextran sulfate in 2× sodium saline citrate (SSC; 20× SSC: 3M sodium chloride, 300 mM trisodium citrate, pH 7.0) was pipetted on a suitable chromosome-containing slide and immediately denatured on a hot plate at 80°C for 2 min. In some experiments, two differentially labeled probes (100 ng of each) were pooled. Hybridization was carried out in a moist chamber at 37°C for 24 h. Post-hybridization washing was performed in 20% formamide in 2× SSC at 42°C. The immunodetection of hapten-labeled probes was performed as described by Mandáková and Lysak (2016b) as follows: biotin-dUTP was detected by avidin–Texas Red (Vector Laboratories) and amplified by goat anti-avidin–biotin (Vector Laboratories) and avidin–Texas Red, and digoxigenin-dUTP was detected by mouse anti-digoxigenin (Jackson ImmunoResearch) and goat anti-mouse–Alexa Fluor 488 (Invitrogen). Chromosomes were counterstained with 4′,6-diamidino-2-phenylindole (DAPI, 2 μg/ml) in Vectashield. The preparations were photographed using a Zeiss Axio Imager 2 epifluorescence microscope with a CoolCube camera (MetaSystems). Images were acquired separately for two or three fluorochromes using appropriate excitation and emission filters (AHF Analysentechnik). Individual monochromatic images were pseudocolored and merged and cropped using Adobe Photoshop CS.

## Results

### *Chamira* and *Heliophila* Have Most Likely Undergone a Shared WGD in Oligocene

Transcriptomes were assembled for *C. circaeoides* and three *Heliophila* species (*H. lactea*, *H.* cf. *longifolia*, and *H. seselifolia*), from which we predicted 16,671 to 30,264 protein-CDS. Compared to the publicly available genome of *H.* aff. *coronopifolia* (Kiefer et al., 2019), which showed 53.2% gene completeness, these transcriptomes had more than 70% of the 1,440 conserved BUSCO genes complete (Supplementary Figure 1). In addition, more than 10% genes were still identified as duplicated ones in all transcriptome-derived CDS after removing potential isoforms (Supplementary Figure 1).

**FIGURE 1:**
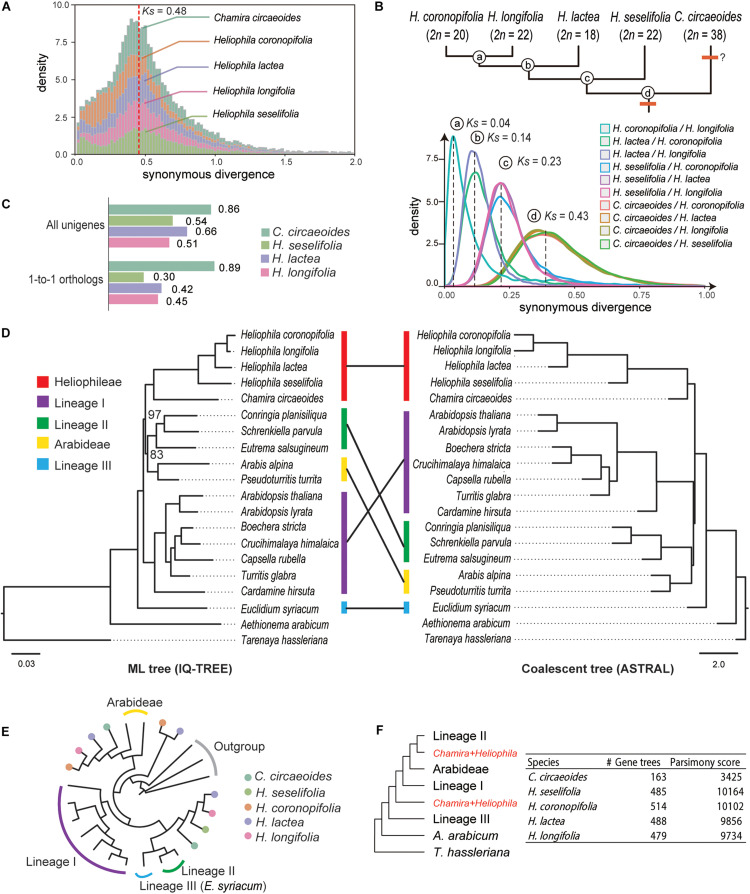
Analyses of synonymous substitutions per synonymous site (*Ks*) and phylogenetic tree topologies. **(A)** *Ks* plots of within-species comparisons in *Chamira* and *Heliophila* species. The red dashed line indicates the position of peaks for the putatively shared WGD event. **(B)** *Ks* plots of between-species comparisons in *Chamira* and *Heliophila* species. Peaks of species divergence are indicated with dashed lines and labeled with circled letters. The upper tree topology shows the branching order of *Chamira* and *Heliophila* species as revealed by *Ks* analyses, with chromosome numbers labeled for each species. **(C)** Comparison of SNP rates in coding sequences of *C. circaeoides*, *H. lactea*, *H.* cf. *longifolia*, and *H. seselifolia*. **(D)** Phylogenetic relationship between *Chamira* + *Heliophila* and other Brassicaceae species using maximum-likelihood (ML) and coalescent approaches. Colored bars indicate different lineages. Unless otherwise mentioned, all relationships received full support in both ML and coalescent trees. Bootstrap values are labeled for two branches that failed to be fully supported in ML analysis. Scale bars below ML and coalescent trees indicate substitution per site and coalescent unit, respectively. **(E)** An example of gene tree topology showing phylogenetic relationship among multicopy genes of *Chamira* and *Heliophila* species. **(F)** The multilabeled tree with the lowest parsimony score, as inferred by gene tree reconciliation analyses in all species. The ASTRAL topology was used as the input of species tree hypothesis.

From within-species comparisons of CDS, we identified 1,711 to 3,838 paralogous gene pairs and calculated their rates of synonymous site changes per synonymous site (*Ks*; Supplementary Table 7). The distribution of *Ks* showed a clear peak between 0.43 and 0.48 in all *Heliophila* species (Figure 1A), which can indicate a lineage-specific mesopolyploidy event as proposed by Mandáková et al. (2012, 2017). Interestingly, a *Ks* peak at the same location was observed in *C. circaeoides* (Figure 1A). To assess whether the WGD event(s) occurred before or after the divergence of *Chamira* and *Heliophila*, we retrieved 7,681 to 16,901 orthologous gene pairs and compared *Ks* peaks from between-species comparisons. We found a *Ks* peak at 0.43 in all comparisons between *Heliophila* species and *C. circaeoides*, which represented the oldest divergence in our comparisons (Figure 1B and Supplementary Table 8). Thus, the WGD event(s) likely occurred before the *Chamira*/*Heliophila* split and might be shared by the two genera. Considering a mutation rate of 8.22 × 10^–9^ substitutions/synonymous site per year (Kagale et al., 2014), the time of WGD or subgenome divergence was estimated between 26.15 and 29.20 Mya, and the *Chamira*/*Heliophila* split around 26.16 Mya.

In addition to the WGD peak, we detected a minor *Ks* peak between 0 and 0.1 in all analyzed species (Figure 1A). By mapping RNA-Seq reads to the assembled transcriptomes, we observed that the heterozygosity in *C. circaeoides* was two times higher than in *Heliophila* species (Figure 1C and Supplementary Table 9). This, along with relatively high chromosome number in *C. circaeoides* (2*n* = 38), may suggest that the minor *Ks* peak in this species represents an additional WGD post-dating the *Chamira*–*Heliophila* divergence (Figure 1B).

### Transcriptome Phylogeny Corroborates the Sistership of *Chamira* and *Heliophila* and Suggests Their Allopolyploid Origin

After including 15 available genomes from major Brassicaceae lineages as well as the outgroup *T. hassleriana* (Cleomaceae), we retrieved 37 strictly single-copy genes that are shared by all species. Our phylogenetic analyses corroborated the sistership of *Chamira* and *Heliophila* (Figure 1D). However, maximum-likelihood (IQ-TREE) and coalescent-based (ASTRAL) methods recovered different topologies regarding the placement of *Chamira* + *Heliophila*. Whereas the ML tree suggested that this clade was sister to lineage II + Arabideae, coalescent analysis showed that it had a more ancestral position, being outside of lineage I + lineage II + Arabideae (Figure 1D). We also retrieved 130 homologous gene groups that consisted of mostly single-copy genes in diploid species and multicopy genes in *C. circaeoides* and *Heliophila* species. We observed that *C. circaeoides* and *Heliophila* genes frequently formed sister clades that were sister to different Brassicaceae lineages (see Figure 1E for an example), which suggested that the mesopolyploidy event(s) involved distant hybridization(s). Despite the number of multicopy genes varying across species, gene tree reconciliation analyses focusing on individual species recovered the same source of potential parental genomes for both *Chamira* and *Heliophila* (Figure 1F).

### The Updated ITS Phylogeny Revealed Four Major Clades in *Heliophila*

A Bayesian 50% majority-rule consensus ITS tree (Supplementary Figure 2) was inferred from sequences of 198 *Heliophila* accessions and five outgroup species (Supplementary Table 1). Four major ITS clades were identified in *Heliophila*. The largest clade A contained 88 accessions, clade B 32 accessions, clade C 73 accessions, and clade D grouped only five accessions. Clade D was newly identified as compared to the previous analyses based on a less extensive taxon sampling (Mummenhoff et al., 2005; Mandáková et al., 2012). All major clades were well supported (posterior probability, *pp* ≥ 0.98) except for clade A (*pp* = 0.69). Among the four major clades, clade D was sister to the other three clades; clades A and C showed a sister relationship. *C. circaeoides* was confirmed as the sister genus of *Heliophila*/Heliophileae.

### Dated Plastome Phylogeny Suggested a Middle Miocene Origin of *Heliophila*

**FIGURE 2:**
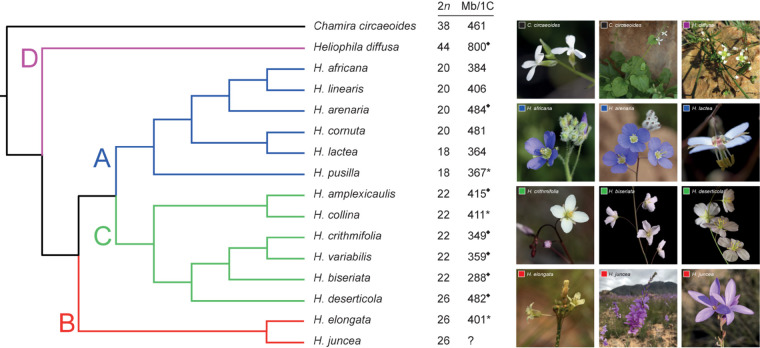
Schematic relationships among the analyzed 15 *Heliophila* species and *C. circaeoides* based on the ITS phylogeny. Capital letters refer to four *Heliophila* clades. Diamond symbols indicate average *C*-values based on estimates for two or more populations; asterisks indicate *C*-values obtained by analysis of partially degraded leaf material. The original ITS phylogeny is presented as Supplementary Figure 2. All photos by TM.

Using the basally resolved ITS tree, 15 *Heliophila* species, proportionally representing the four clades, and *C. circaeoides* were selected for lcWGS (Figure 2 and Supplementary Table 2). The next-generation sequence data was used to construct whole-plastome phylogeny and analyze nuclear repeatomes of the 16 genomes.

We assembled complete cp genomes for all 16 sequenced species, ranging in length from 152,794 (*C. circaeoides*) to 154,300 bp (*H. pusilla* var. *pusilla*) (Supplementary Table 10). All the cp genomes showed a typical quadripartite structure in which a large single-copy (LSC) region (82,736–83,766 bp) and a short single-copy (SSC) region (17,429–17,958 bp) are separated by two inverted repeat (IR) copies (26,235–26,413 bp). All analyzed genomes encoded 131 genes, including 86 protein-coding genes, 37 tRNA genes, and eight rRNA genes (Supplementary Table 10). The GC content of the assembled cp genomes ranged between 36.1 and 36.7%.

After excluding unalignable or ambiguous regions and sites, a supermatrix with 100,707 nucleotide sites was generated, of which 15,637 (15.5%) were parsimony informative. *Heliophila* species were retrieved as a monophyletic clade sister to *C. circaeoides*. The maternal phylogeny was largely congruent with the above-described ITS phylogeny, except for *H. diffusa* var. *diffusa* (clade D) clustering with clade B species (Supplementary Figure 3). Based on the plastome phylogeny, we estimated that the split between (Heliophileae + *Chamira*) species and their closest relative, *S. aquatica*, occurred (15.95) 20.26 (24.64) Mya, at the Oligocene–Miocene boundary. The divergence between *Chamira* and *Heliophila* was dated to (13.77) 18.53 (23.33) Mya, followed by the diversification of the four *Heliophila* clades c. 16 to 8 Mya.

### Repeatome Analysis

**FIGURE 3:**
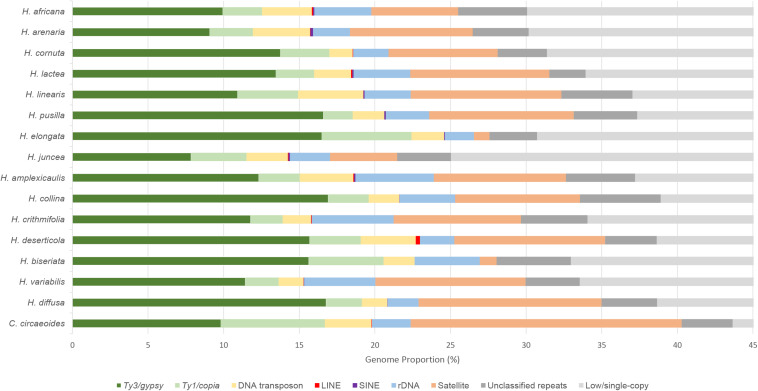
Relative repeat abundances and low/single-copy sequences identified in sequenced genomes of 15 *Heliophila* species and *C. circaeoides*. Low/single-copy sequences above 45% were discarded.

The RepeatExplorer2 pipeline was used to analyze and compare the repeatomes of 15 *Heliophila* species and *C. circaeoides*. Maximum number of reads was used for the detailed repeatome analysis with the genome coverage from 0.16× to 0.47× (Table 1). The total repeat content of the analyzed species ranged from 25.04% to 43.66%, whereas single- or low-copy sequences made up the remainder of the genome sequences (Figure 3). In all *Heliophila* genomes, the predominant repeat type was long terminal repeat (LTR) retrotransposons, ranging from 11.51% (*H. juncea*) to 22.42% (*H. elongata*) (Table 2 and Figure 3). The most abundant repeat type of the *C. circaeoides* genome was tandem repeats (17.92%), whereas among the 15 *Heliophila* genomes, tandem repeat abundances varied from 1.03% (*H. elongata*) to 12.10% (*H. diffusa*) (Table 2 and Figure 3). In all the analyzed genomes, DNA transposon abundances were lower compared with LTR retrotransposons, ranging from 1.54% (*H. cornuta* var. *cornuta*) to 4.31% (*H. linearis* var. *linearis*) (Table 2 and Figure 3).

**TABLE 1:** Characteristics of NGS data used for repeatome analysis.

Species	Clade	Total repeats (%)	No. of reads	Genome coverage	No. of clusters
*Heliophila africana*	A	30.07	511,217	0.19×	232
*H. arenaria* subsp. *arenaria*	A	30.18	901,412	0.26×	183
*H. cornuta* var. *cornuta*	A	31.38	652,717	0.19×	203
*H. lactea*	A	33.93	744,973	0.29×	177
*H. linearis* var. *linearis*	A	37.03	1,002,030	0.35×	209
*H. pusilla* var. *pusilla*	A	37.34	576,080	0.22×	164
*H. elongata*	B	30.73	662,141	0.23×	181
*H. juncea*	B	25.04	886,973	0.31×	173
*H. amplexicaulis*	C	37.21	961,390	0.32×	165
*H. collina*	C	38.90	774,496	0.31×	130
*H. crithmifolia*	C	34.06	853,487	0.29×	224
*H. deserticola* var. *micrantha*	C	38.64	1,304,122	0.38×	281
*H. biseriata*	C	32.96	962,154	0.47×	276
*H. variabilis*	C	33.55	1,056,742	0.41×	148
*H. diffusa* var. *diffusa*	D	38.67	911,217	0.16×	141
*Chamira circaeoides*	–	43.66	690,569	0.21×	163

**TABLE 2:** Detailed classification of repetitive elements and their genome proportions (%).

		Clade															
		A						B		C						D	
Repeat family		HeAfr	HeAre	HeCor	HeLac	HeLin	HePus	HeElo	HeJun	HeAmp	HeCol	HeCri	HeDes	HeBis	HeVar	HeDif	ChCir
LTR retrotransposons		12.54	11.93	17.00	15.99	14.93	18.54	22.42	11.51	15.03	19.59	13.90	19.07	20.58	13.63	19.14	16.69
*Ty3/gypsy*	*Athila*	7.62	8.21	8.23	10.81	6.95	13.99	12.79	5.87	9.53	11.56	7.67	6.38	9.36	8.13	12.77	6.57
	*CRM*	0.75	0.45	1.56	1.43	2.15	1.64	1.85	1.63	2.26	4.51	0.88	5.31	1.76	1.12	2.94	2.27
	*Tekay*	0.84	0.28	3.60	0.75	1.78	0.67	0.54	0.07	0.39	0.78	3.07	3.72	3.58	2.13	0.59	0.00
	*Galadriel*	0.06	0.00	0.16	0.26	0.00	0.00	0.10	0.01	0.04	0.00	0.07	0.12	0.31	0.00	0.00	0.48
	*Reina*	0.02	0.02	0.03	0.00	0.00	0.01	0.00	0.00	0.00	0.00	0.06	0.02	0.24	0.00	0.00	0.29
	*Ogre/Tat*	0.00	0.00	0.04	0.00	0.00	0.17	0.32	0.17	0.00	0.00	0.00	0.09	0.25	0.00	0.45	0.00
	Unclassified	0.66	0.14	0.15	0.23	0.03	0.12	0.90	0.10	0.10	0.06	0.03	0.06	0.12	0.05	0.03	0.21
	Total	9.94	9.09	13.76	13.47	10.92	16.60	16.50	7.84	12.32	16.91	11.77	15.69	15.63	11.43	16.78	9.82
*Ty1/copia*	*Ale*	0.21	0.20	0.14	0.11	0.35	0.21	1.43	0.81	0.10	0.22	0.44	0.54	1.55	0.17	0.30	3.31
	*Bianca*	1.14	1.26	1.86	0.67	1.29	1.28	2.35	0.76	0.96	1.20	1.26	1.06	1.28	1.17	0.69	1.23
	*Angela*	0.27	0.34	0.04	0.18	0.39	0.13	0.25	0.18	0.13	0.33	0.03	0.35	0.34	0.11	0.03	0.07
	*Ivana*	0.29	0.36	0.20	0.84	0.55	0.09	0.85	0.21	0.64	0.31	0.09	0.31	0.10	0.24	0.86	0.66
	*TAR*	0.40	0.45	0.40	0.33	0.79	0.10	0.29	0.42	0.41	0.51	0.12	0.43	0.53	0.22	0.01	1.14
	*Tork*	0.23	0.15	0.56	0.31	0.59	0.10	0.67	1.15	0.40	0.05	0.16	0.63	0.93	0.18	0.16	0.40
	*SIRE*	0.05	0.04	0.03	0.08	0.03	0.01	0.04	0.10	0.02	0.03	0.01	0.05	0.13	0.09	0.24	0.04
	Unclassified	0.01	0.03	0.01	0.01	0.02	0.03	0.03	0.04	0.05	0.04	0.01	0.01	0.08	0.01	0.06	0.01
	Total	2.60	2.84	3.24	2.52	4.00	1.94	5.92	3.67	2.71	2.68	2.12	3.37	4.95	2.20	2.36	6.87
DNA transposons	*Harbinger*	0.53	0.58	0.04	0.17	0.38	0.11	0.08	0.04	0.23	0.07	0.05	0.11	0.21	0.03	0.06	0.19
	*Helitron*	0.38	0.36	0.04	0.12	0.11	0.10	0.24	0.17	0.42	0.28	0.24	0.20	0.11	0.05	0.09	0.00
	*CACTA*	0.67	0.56	0.57	0.49	1.05	0.35	0.53	0.48	0.96	0.60	0.17	1.61	0.48	0.38	0.29	1.85
	*Mariner*	0.14	0.15	0.00	0.18	0.15	0.09	0.00	0.01	0.07	0.05	0.07	0.07	0.05	0.03	0.18	0.00
	*Mutator*	0.45	0.82	0.50	0.59	0.92	0.91	0.86	1.12	0.88	0.51	0.42	0.86	0.87	0.60	0.72	0.43
	*hAT*	0.81	0.61	0.08	0.43	0.89	0.24	0.23	0.45	0.82	0.11	0.24	0.59	0.17	0.23	0.11	0.50
	Unclassified	0.33	0.72	0.31	0.45	0.81	0.28	0.22	0.46	0.17	0.40	0.69	0.20	0.17	0.34	0.22	0.12
	Total	3.29	3.79	1.54	2.44	4.31	2.08	2.16	2.74	3.54	2.02	1.88	3.64	2.06	1.65	1.69	3.09
LINE		0.08	0.02	0.03	0.07	0.01	0.00	0.00	0.05	0.05	0.01	0.04	0.28	0.00	0.02	0.00	0.03
SINE		0.08	0.17	0.00	0.09	0.07	0.10	0.06	0.09	0.09	0.00	0.00	0.00	0.00	0.02	0.01	0.00
rDNA		3.76	2.45	2.34	3.75	3.05	2.87	1.91	2.65	5.18	3.69	5.42	2.27	4.31	4.71	2.05	2.56
Tandem repeats		5.75	8.12	7.21	9.19	9.96	9.56	1.03	4.44	8.74	8.25	8.42	9.97	1.11	9.93	12.10	17.92
Unclassified repeats		4.56	3.70	3.26	2.40	4.70	4.18	3.16	3.56	4.57	5.34	4.40	3.42	4.91	3.58	3.67	3.37
Low/single-copy sequences		69.93	69.82	68.62	66.07	62.97	62.66	69.27	74.96	62.79	61.10	65.94	61.36	67.04	66.45	61.33	56.34
All repeats total		30.07	30.18	31.38	33.93	37.03	37.34	30.73	25.04	37.21	38.90	34.06	38.64	32.96	33.55	38.67	43.66

*HeAfr, Heliophila africana; HeAre, Heliophila arenaria; HeCor, Heliophila cornuta; HeLac, Heliophila lactea; HeLin, Heliophila linearis; HePus, Heliophila pusilla; HeElo, Heliophila elongata; HeJun, Heliophila juncea; HeAmp, Heliophila amplexicaulis; HeCol, Heliophila collina; HeCri, Heliophila crithmifolia; HeDes, Heliophila deserticola; HeBis, Heliophila biseriata; HeVar, Heliophila variabilis; HeDif, Heliophila diffusa; ChCir, Chamira circaeoides.*

We tested possible correlations between the estimated abundances of identified repeat families (Table 2) and genome size (Mb/1C) of the analyzed *Heliophila* species. The total repeat content was positively correlated with genome size (*p* value = 0.0006, *R*^2^ = 0.6446). A weak but significant positive correlation was found between tandem repeat content and genome size (*p* value = 0.0397, *R*^2^ = 0.3071).

### Transposable Elements

*Ty3/gypsy* was the most abundant superfamily of LTR retrotransposons in *Heliophila* species, ranging from 7.84% (*H. juncea*) to 16.91% (*H. collina*), while *Ty1/copia* retrotransposons were less prominent, ranging from 1.94% (*H. pusilla*) to 5.92% (*H. elongata*). In the *C. circaeoides* genome, LTR retrotransposons represent 16.69% of the genome (9.82% of *Ty3/gypsy* and 6.87% of *Ty1/copia* elements) (Figure 3).

Analyzing the *Ty3/gypsy* superfamily, Chromovirus-type elements were represented by *CRM*, *Tekay*, *Galadriel*, and *Reina* lineages (ordered by their abundances), whereas non-Chromovirus-type elements were represented by *Athila* and *Ogre/Tat* lineages (Table 2). In all analyzed genomes, *Athila* was the predominant lineage. The abundance of *Athila* elements ranged from 5.87% in *H. juncea* to 13.99% in *H. pusilla*. From Chromovirus lineage elements, *CRM* was found to be the most abundant, ranging from 0.45% in *H. arenaria* subsp. *arenaria* to 5.31% in *H. deserticola* var. *micrantha*. In *C. circaeoides*, *Athila* lineage was the most abundant *Ty3/gypsy* element (6.57%), followed by *CRM* (2.27%).

*Ty1/copia* superfamily consisted of seven lineages: *Bianca*, *Ale*, *Tork*, *TAR*, *Ivana*, *Angela*, and *SIRE* (ordered by their abundances) (Table 2). *Bianca* was identified as the most abundant lineage among the *Heliophila* species, ranging from 0.67% in *H. lactea* to 2.35% in *H. elongata*. In *C. circaeoides*, *Ale* lineage was the most abundant *Ty1/copia* element (3.31%), followed by *Bianca* (1.23%). The amplification of the *Ale* elements differentiated the *C. circaeoides* genome from those of *Heliophila* species. The diversity and abundances of the identified LTR retrotransposons have not followed the infrageneric groupings in *Heliophila*, and LTR retroelement abundance (in Mb) has not been found correlated with genome size (*p* value = 0.0569, *R*^2^ = 0.2700).

Non-LTR retrotransposons, *LINE* and *SINE* elements, were found at very low abundances or not detected in the 16 analyzed genomes; the highest abundances were encountered in *H. deserticola* (0.28%) for *LINE* and *H. arenaria* (0.17%) for *SINE* (Table 2).

In *Heliophila* species, DNA transposons were represented by *Mutator*, *CACTA*, *hAT*, *Helitron*, *Harbinger*, and *Mariner* lineages (Table 2). *Mutator* (0.42% in *H. crithmifolia* to 1.12% in *H. juncea*) and *CACTA* (0.17% in *H. crithmifolia* to 1.61% in *H. deserticola*) were the more abundant elements. In *C. circaeoides*, *CACTA* lineage was the most abundant DNA transposon (1.85%), followed by *hAT* (0.50%). The diversity and abundance of DNA transposons did not correspond to the infrageneric *Heliophila* clades, but the amounts of identified DNA transposons (in Mb) were found to be weakly correlated with genome size (*p* value = 0.0184, *R*^2^ = 0.3823).

### Tandem Repeats

In total, 124 tandem repeats were identified in the analyzed *Heliophila* and *Chamira* genomes. The identified tandem repeats varied in monomer lengths (e.g., 27-bp HeJun6 in *H. juncea* and 4,034-bp ChCir9 in *C. circaeoides*), numbers (four in *H. pusilla* and *H. elongata* up to 16 in *C. circaeoides*), and abundances (from 1.03% in *H. elongata* to 17.92% in *C. circaeoides*) (Supplementary Table 11). The high tandem repeat content in *H. diffusa* (12.10%) differentiates this genome from genomes of the other three *Heliophila* clades. Tandem repeats of species in clades A, B, and C ranged from 4.44% to 9.97%, except for ∼1% in *H. elongata* (clade B) and *H. biseriata* (clade C) (Figure 3).

No apparent correspondence between the diversity of tandem repeats and their genomic proportion was observed. For example, 10 tandem repeats identified in *H. biseriata* represented only 1.11% of its genome, whereas only four tandem repeats built up 9.56% of the *H. pusilla* genome (Supplementary Table 11). In all *Heliophila* species, one or two tandem repeats were dominating their tandem repeatomes [e.g., HeAfr1: 4.72% (out of 5.75%), HeAmp1: 5.9% (8.74%), HeJun1: 2.1% and HeJun2: 1.73% (4.44%)].

The genome of *C. circaeoides* exhibited the highest number of identified tandem repeats among all the sequenced species. The monomer length of the 16 tandem repeats varied from 180 to 4,034 bp, whereby seven and four repeats were longer than 1,000 and 3,000 bp, respectively (Supplementary Table 11). The abundances of these repeats ranged from 0.074% to 0.59%. Seven tandem repeats with monomers longer than 1,000 bp were also identified in four *Heliophila* genomes from sister clades A and C species (*H. africana*, *H. biseriata*, *H. crithmifolia*, and *H. linearis*) at very low abundances (<0.1%, except for HeBis2: 0.24%).

### Shared Tandem Repeats

**FIGURE 4:**
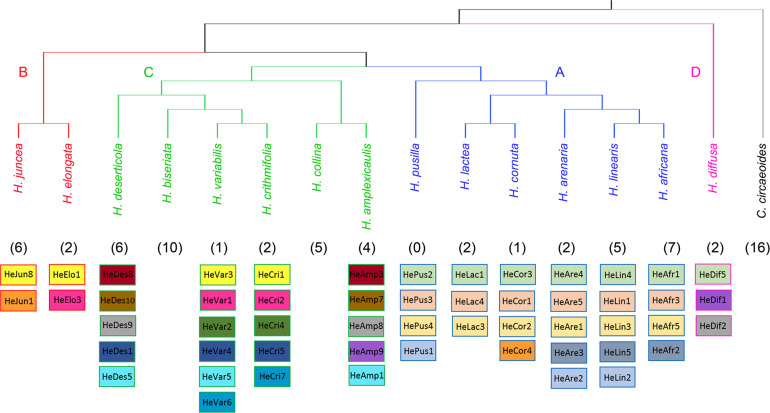
Overview of shared tandem repeats among the 15 *Heliophila* species analyzed. The ITS tree (Supplementary Figure 2) was used to display species relationships. Shared tandem repeats are color-coded; numbers of species-specific tandem repeats are indicated in parenthesis.

Our analyses have not identified any homologous tandem repeats between *C. circaeoides* and *Heliophila* species. In *Heliophila* genomes, among the 108 tandem repeats identified, 16 repeats were found to be shared among two or more species (Figure 4 and Supplementary Table 11). Monomer lengths of the shared tandem repeats varied between 158 and 184 bp, and overall pairwise sequence homologies ranged from 82.5% to 100% (Supplementary Tables 11,12). Dot-plot comparison of consensus monomer sequences of shared tandem repeats is shown in Supplementary Figure 4, and multiple and pairwise alignments of the 16 shared repeats are presented in Supplementary Figure 5.

In clade A species, three tandem repeats were shared among all the six genomes, whereas one repeat was shared only by three species of the *H. africana* subclade (HeAfr2, HeAre3, and HeLin5). Whereas five clade A species have unique tandem repeats, all four tandem repeats detected in *H. pusilla* were shared either among all clade A species (HePus2, HePus3, and HePus4) or only with *H. arenaria* (HeAre2) and *H. linearis* (HeLin2). Interestingly, the 168-bp HeCor4 repeat in *H. cornuta* was found to be homologous to the HeJun1 in *H. juncea* from clade B (Figure 4 and Supplementary Tables 11,12).

In clade B, *H. elongata* and *H. juncea* shared one tandem repeat (HeElo1 and HeJun8) which was also shared with two clade C species – *H. crithmifolia* (HeCri1) and *H. variabilis* (HeVar3). The 184-bp HeElo3 identified in the *H. elongata* genome was also detected in *H. crithmifolia* (HeCri2) and *H. variabilis* (HeVar1) (Figure 4 and Supplementary Tables 11,12). Among the six clade C genomes analyzed, two genomes (*H. biseriata* and *H. collina*) possessed only species-specific repeats, while 10 repeats were shared by at least two of the four remaining species. Sister species *H. crithmifolia* and *H. variabilis* shared five different repeats, whereby two were also shared by *H. deserticola* (HeDes1, HeDes5) and the other two were identified in two clade B species (see above). Three other repeats were shared between *H. amplexicaulis* (HeAmp3, HeAmp7, and HeAmp8) and *H. deserticola* (HeDes8, HeDes9, and HeDes10) species without a sister relationship (Figure 4 and Supplementary Tables 11,12). *H. diffusa* shared one repeat (HeDif5) with all clade A genomes and two repeats (HeDif1, HeDif2) with clade C species *H. amplexicaulis* (HeAmp8, HeAmp9) and *H. deserticola* (HeDes9) (Figure 4 and Supplementary Tables 11,12).

In summary, the identified tandem repeats shared among *Heliophila* species, but not with *Chamira*, corroborates the monophyletic origin of the former genus. The three repeats shared among all clade A genomes may reflect younger age of speciation events in this group (Supplementary Figure 3). Tandem repeatomes in clade C genomes show high evolutionary dynamism, manifested by (i) high diversity of shared satellites, (ii) some repeats being shared with more ancestral clades B and D, and (iii) accelerated evolution or elimination of shared repeats in two species (*H. biseriata* and *H. collina*).

### Phylogenetic Analysis of the Identified Repeats

**FIGURE 5:**
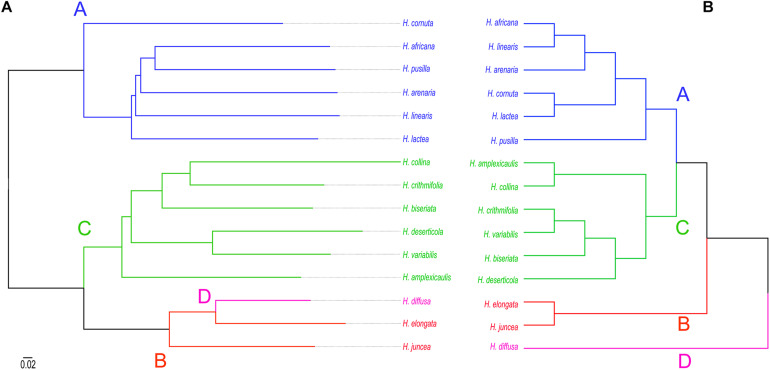
Phylogenetic relationships between 15 *Heliophila* species based on repeat sequence similarities **(A)** and ITS sequences **(B)**. Scale bar indicates mean branch length across all individual trees to infer the consensus tree.

Consensus tree phylogeny was reconstructed using the ape package (Paradis and Schliep, 2019) in R based on pairwise genetic distances between all repeats in the 100 most abundant clusters retrieved from RepeatExplorer2 comparative clustering analysis. In the dataset which consisted of 15 *Heliophila* species, 25 clusters included sequence overlaps (similarities) between reads from all species to generate sequence similarity matrices. In 75 clusters, sequence reads shared by all the analyzed species were lacking, indicating that those repeats are either species- or clade-specific. The consensus tree, reconstructed from 25 clusters with complete similarity matrices, separated repeatomes of clade A and C species, whereas clade B and D genomes formed a third clade (Figure 5A). This topology is congruent with the plastome (Supplementary Figure 2) and ITS tree (Figure 5B) in retrieving clades A and C, but differs by grouping clade B and D genomes into one clade. To test whether the number of retrieved similarity-based clusters may change, two alternative sub-datasets with either clade A or clade C genomes excluded were analyzed. The exclusion of either clade C or clade A genomes resulted in the separation of *H. diffusa* from the two clade B species (Supplementary Figure 6), similar to the ITS-based phylogeny (Figure 5B and Supplementary Figure 2). While the repeat-based analysis identified three major infrageneric clades in *Heliophila* (Figure 5A), the interspecies relationships in clades A and C differed from those in the ITS-based tree (Figure 5B) and plastome phylogeny (Supplementary Figure 3).

RepeatExplorer2 comparative analysis read abundance matrix was transformed to distance matrix and used to reconstruct the hierarchical clustering relationship of *Heliophila* species and *Chamira* (Supplementary Figure 7). Clade A, B, and C species formed separated clusters in the reconstructed dendrogram, and clade D species *H. diffusa* together with *C. circaeoides* was retrieved as sister to the remaining *Heliophila* genomes. This clustering was incongruent with interclade relationships in the plastome (Supplementary Figure 3), ITS, and repeat sequence similarity-based (Figure 5) phylogenies.

### Chromosomal Localization of the Identified Repeats

**FIGURE 6:**
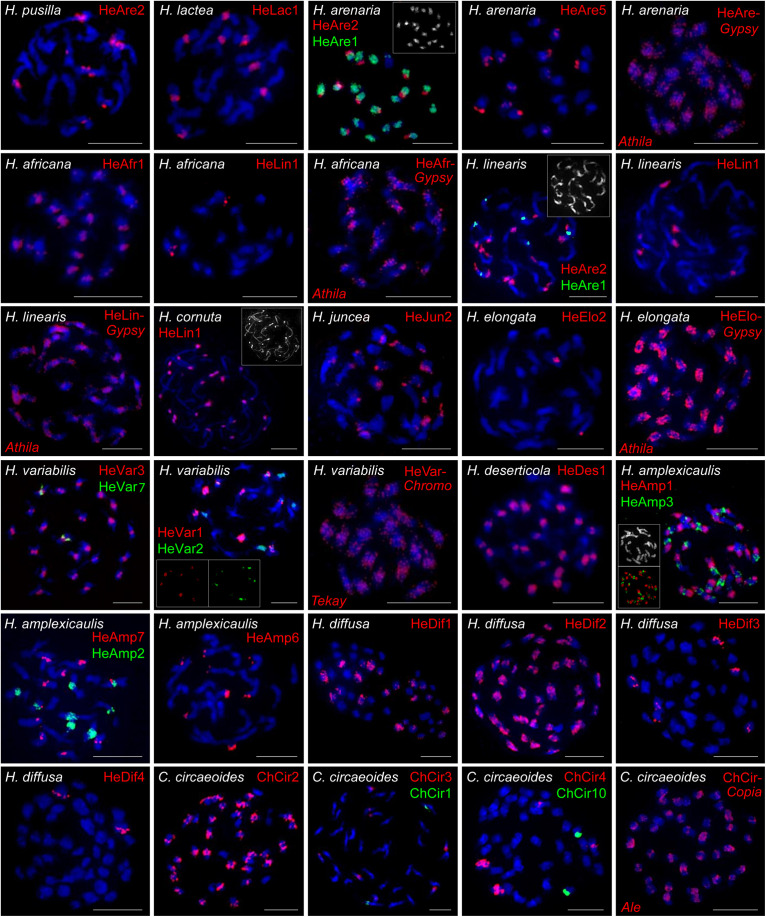
FISH localization of the selected tandem repeats and retroelements on mitotic metaphase chromosomes of *Heliophila* species and *C. circaeoides*. Chromosomes were counterstained by DAPI; FISH signals are shown in color as indicated. Detailed information on the localized repeats is provided in Supplementary Table 6. Scale bars, 10 μm.

Chromosomal distribution of selected identified repeats was determined by fluorescence *in situ* hybridization (FISH) in six clade A species (*H. africana*, *H. arenaria*, *H. cornuta*, *H. lactea*, *H. linearis*, *H. pusilla*), two clade B species (*H. elongata*, *H. juncea*), three clade C species (*H. amplexicaulis*, *H. deserticola*, *H. variabilis*), in clade D species *H. diffusa*, and *C. circaeoides* (Supplementary Table 6 and Figure 6).

In clade A, FISH of DNA probe for the 172-bp repeat HeAre2 identified species-specific chromosomal distribution of the satellite in three *Heliophila* genomes. HeAre2 was identified in pericentromeric heterochromatin of four chromosome pairs in *H. pusilla*, subtelomeric region of five chromosome pairs in *H. arenaria*, and at terminal heterochromatic knobs of seven chromosome pairs in *H. linearis*. In *H. arenaria* and *H. linearis*, the 174-bp HeAre1 repeat localized to pericentromeric regions of all and three chromosome pairs, respectively. In *H. africana*, *H. cornuta*, and *H. linearis*, the 171-bp HeLin1 tandem repeat showed localization at one, all (c. 24), and three chromosome pairs, respectively. In *H. lactea*, the 177-bp HeLac1 tandem repeats localized to pericentromeric regions of four chromosome pairs. The 177-bp HeAfr1 and 171-bp HeAre5 tandem repeats localized to all pericentromeres in *H. africana* and six chromosome termini in *H. arenaria*, respectively (Figure 6). In clade B genomes, the 167-bp HeJun2 and 383-bp HeElo2 repeats were present in subtelomeric regions of c. 20 chromosomes in *H. juncea* and pericentromeres of one chromosome pair in *H. elongata*, respectively (Figure 6).

In clade C species *H. variabilis*, four major tandem repeats formed pericentromeric chromatin. The 177-bp HeVar3 repeat localized to all chromosome pairs, the 168-bp HeVar2 provided hybridization signals on five chromosome pairs, the 184-bp HeVar1 localized to four chromosome pairs, and the 832-bp HeVar7 repeat gave hybridization signal on one chromosome pair. The 178-bp HeDes1 tandem repeat was located at all but two pericentromeres in *H. deserticola*. In *H. amplexicaulis*, 172-bp HeAmp2, 175-bp HeAmp3, and 184-bp HeAmp7 tandem repeats localized to pericentromeric heterochromatin of four, 11, and five chromosome pairs. The 188-bp HeAmp6 tandem repeat localized to subtelomeric regions of four chromosome pairs. Finally, the 162-bp HeAmp1 provided a strong hybridization signal at all interstitial and terminal heterochromatic knobs (Figure 6).

In clade D species *H. diffusa*, four major tandem repeats formed pericentromeres. The 177-bp HeDif2, 178-bp HeDif1, 184-bp HeDif3, and 171-bp HeDif4 repeats gave hybridization signals in all 22, c. 11, three, and one chromosome pair, respectively (Figure 6).

In *C. circaeoides*, three pericentromeric (294-bp ChCir2, 202-bp ChCir3, and 198-bp ChCir4) and two subtelomeric (249-bp ChCir1 and 1,427-bp ChCir10) tandem repeats were localized. The ChCir2 repeat was present in all pericentromeres, whereas ChCir3 and ChCir4 localized in centromeres to four and two chromosome pairs, respectively. ChCir1 and ChCir10 showed localization at chromosome termini of two different chromosome pairs (Figure 6).

In the investigated *Heliophila* and *Chamira* species, retrotransposons were mostly accumulated in pericentromeric heterochromatin; however, to a lesser extent, they were also distributed on chromosome arms (distribution of *Ty3/gypsy* in *H. africana*, *H. arenaria* subsp. *arenaria*, *H. linearis*, *H. elongata*, and *H. variabilis* and of *Ty1/copia* in *C. circaeoides* are shown in Figure 6).

### Repeatome of *C. circaeoides* vs. Repeatomes of *Heliophila* Species

Detailed repeat analysis showed that *C. circaeoides* contained about 5% more repetitive elements (43.66%) in its genome compared with *H. diffusa* which exhibited the highest repeat content (38.67%) among *Heliophila* genomes (Figure 3), despite the genome size difference between the two species (461 and 800 Mb, respectively, Figure 2).

*C. circaeoides* showed minor differences in its overall repeatome composition compared with the 15 *Heliophila* species analyzed (Figure 3). Total LTR retrotransposon abundances in *C. circaeoides* were comparable with those observed in *Heliophila* genomes (16.69% in *C. circaeoides* vs. 11.51% to 22.42% in *Heliophila*, Table 2). Whereas *Ty3/gypsy* abundance was similar in *Chamira* and *Heliophila* genomes, *Ty1/copia* abundance in *C. circaeoides* was observed to be the highest (6.87%) among all the sequenced genomes (the highest proportion of *Ty1/copia* elements was detected in *H. elongata* – 5.92%). Unlike in *Heliophila* species, where *Bianca* is the predominant *Ty1/copia* lineage, *Ale* lineage was the most abundant *Ty1/copia* element in *C. circaeoides* (3.31%) (Table 2).

The most distinct feature of the *C. circaeoides* genome is the high accumulation of tandem repeats (17.92%, Figure 3). In *Heliophila*, the highest genomic proportion of tandem repeats was found in *H. diffusa* (12.10%). In contrast to *Heliophila* genomes, long monomer satellites constitute a significant portion of the *C. circaeoides* tandem repeatome, such as ChCir5: 3,388 bp – 0.59%, ChCir6: 3,342 bp – 0.42%, ChCir7: 3,558 bp – 0.37%, and ChCir9: 4,034 bp – 0.17% (Supplementary Table 11).

## Discussion

### The Origin of *Chamira* and *Heliophila* Was Preceded by a WGD

By analyzing transcriptomes of four *Heliophila* species (Mandáková et al., 2017; and this study), we corroborated the earlier conclusion based on chromosome painting data that the genus has undergone a mesopolyploid WGD (Mandáková et al., 2012, 2017). The occurrence of similarly positioned *Ks* peaks in *Heliophila* and *Chamira* genomes, along with the repeatedly retrieved sister relationship of both genera and their sympatry in the Greater Cape Floristic Region, suggests that either the WGD predated the *Chamira*/*Heliophila* divergence or the ancestors of both mesopolyploid genera were closely related. Whereas transcriptome-based divergence time estimates dated the WGD between 26 and 29 Mya and the *Chamira*/*Heliophila* split to 26 Mya, plastome-based dating yielded somewhat younger dates of the *Chamira*/*Heliophila* divergence (c. 21 Mya) and dated the diversification of the four *Heliophila* clades to c. 14–16 Mya. Nevertheless, transcriptome as well as plastome data congruently date the WGD to Oligocene or Miocene and major infrageneric cladogenesis in *Heliophila* to Middle Miocene. Although a much younger origin of *Heliophila* was previously proposed by Mandáková et al. (2012), we reason that those estimates were affected by the use of questionable fossil records and secondary calibration points (Franzke et al., 2016).

Chromosome number of 2*n* = 38 established for *Chamira* (Mandáková et al., 2015; and this study) is similar to those of *Heliophila* neopolyploids (2*n* = 32, 36, 40, 44, 60, 64, 80, and 88; Mandáková et al., 2012) and suggests that the mesopolyploid WGD might have been followed by an additional genome duplication in *Chamira*. Only genome sequences of *C. circaeoides* can shed more light into its genome history and phylogenomic relationship to *Heliophila*. Despite the overall rarity of (neo)polyploidy in Cape flora (Oberlander et al., 2016), ancient WGDs, such as that documented in *Chamira* and *Heliophila*, are probably awaiting their discovery in other southern African angiosperm lineages.

### Major Clades of the *Heliophila* Phylogeny

The monophyly of *Heliophila* and its sister position to *Chamira* were established by Mummenhoff et al. (2005) based on analysis of rDNA ITS sequences. That study retrieved three main clades in *Heliophila* which were confirmed as a basal trichotomy in a follow-up ITS-based study including more species (Mandáková et al., 2012). Herein, by further expanding our taxon sampling, we recovered four well-resolved ITS clades, with clade D (*H. diffusa*, *H. pendula*, and a putative hybrid, aff. *H. macra*) being sister to the three remaining clades (Supplementary Figure 2). The plastome phylogeny was largely congruent with the ITS tree, although it indicated a closer relationship between clades D and B (Supplementary Figure 3). The overall congruence between the two phylogenies further corroborates ITS as a reliable marker for inferring infrageneric relationships in *Heliophila* and other eukaryotic lineages (e.g., García-Robledo et al., 2013; Wang et al., 2015; Minamoto et al., 2017; Yang et al., 2018).

Future analyses of more unplaced species (particularly the *H. concatenata* species complex, *H. astyla*, *H. meyeri*, *H. obibensis*, *H. patens*, *H. scandens*, and some undescribed species) should clarify whether the basal D group could be expanded or whether further clades will be revealed. Altogether, ITS and plastome phylogenies corroborated a minimum of three to four major clades in *Heliophila*. At least two major clades (corresponding to ITS clades A+D and clades B+C, respectively) were retrieved based on pollen types (Kumwenda, 2003), and chromosome number variation in *Heliophila* also supports such cladogenesis. The chromosome number 2*n* = 44 is repeated in the two most morphologically related species of clade D and is rare elsewhere. While species of clade A have mostly chromosome number of 2*n* = 20, 2*n* = 22 is prevalent in clade C species. The known chromosome numbers of B clade species are more variable (2*n* = 16, 22, 26, 32, and 64; *H. dregeana*, *H. elongata*, and *H. juncea*; Mandáková et al., 2012 and this study). This pattern is congruent with the sister relationship of clades A and C, as well as with the more ancestral position of clade B in both nuclear and plastome phylogenies (Supplementary Figures 2,3). While the occurrence of truly monocarpic species is limited to clades A, C, and D, the apparent woodiness and the presence of an intercalary type of inflorescence even in short-lived perennial species are diagnostic characters of clade B species.

### Repeatome Diversity Is Reflecting Infrageneric Cladogenesis in *Heliophila*

A substantial fraction of nuclear plant genomes is composed of repeated DNA. These highly abundant genomic elements are influencing the function and evolution of plant genomes (e.g., Macas et al., 2011; Garrido-Ramos, 2015, 2017), and their diversity and abundance patterns can reflect phylogenetic distances (Dodsworth et al., 2014, 2017; Bolsheva et al., 2019; Vitales et al., 2020). Here, we sequenced and analyzed repetitive elements of 15 *Heliophila* species proportionally representing four major infrageneric clades. As transposable elements (TEs) are usually conserved across closely related species groups (Moisy et al., 2014; Wicker et al., 2018), we did not expect to identify clade-specific TEs in *Heliophila*. TAREAN analysis detected 108 tandem repeats in the sequenced *Heliophila* genomes. Fifty-four percent of all tandem repeats identified in *Heliophila* had a monomer length between 170 and 190 bp; the remaining 46% ranged widely in length from 27 to 2,012 bp in *H. juncea* and *H. biseriata*, respectively. Out of the 108 tandem repeats, 56 (51.9%) were species-specific, 32 (29.6%) shared among species of the same clade, and 20 (18.5%) were shared across the clades.

Most within-clade shared repeats were identified among clade A species – 43% of the repeats were shared among all six species and 14.3% were shared among three species of the clade. Tandem repeatomes of clade C species are more divergent, with 12% of shared repeats being shared among three out of six species analyzed and 31% of repeats shared by only two species. The two clade B species share only a single tandem repeat (one out of 12 identified). No shared repeats were found between any clade A and clade C species, and only 4 tandem repeats were homologous between clade B and clade D species.

According to the dated plastome phylogeny (Supplementary Figure 3), clades B+D split from clades A+C ∼12 Mya and all four clades diverged between 10 and 11 Mya; based on the ITS tree (Supplementary Figure 2), clade D was the first to diverge from the remaining three clades. While the major diversification within clades A and C occurred around 7 Mya, a number of speciation events in clade A seem to be younger than species diversifications in clade C (though the phylogeny suffers from species under-representation). As tandem repeats are evolving rapidly in most cases (Henikoff et al., 2001; Melters et al., 2013), their sequence conservation can be observed only on short evolutionary distances (Henikoff et al., 2001; Meraldi et al., 2006; Koukalova et al., 2010; Renny-Byfield et al., 2013; Dodsworth et al., 2014). Hence, the highest number of shared repeats among clade A species may reflect their close relationships and more recent origins. Similarly, in clade C, the highest number of shared repeats was identified in the species pair *H. crithmifolia*–*H. variabilis* representing the youngest (3.4 Mya) speciation within this clade. Some identified tandem repeats had a relic character, linking distantly related lineages, such as the two repeats shared between *H. elongata* and *H. juncea* from clade B and *H. crithmifolia* and *H. variabilis* from clade C. The most basal species *H. diffusa* (clade D) shares one repeat (HeDif5) with all clade A genomes and two with clade C species (HeDif1: *H. amplexicaulis*, HeDif2: *H. amplexicaulis* and *H. deserticola*). The three repeats shared between clade C and D genomes, and the only tandem repeat (HeCor4) shared between clade A (*H. cornuta*) and clade B (*H. juncea*) remained conserved for 12 million years since the divergence of these clades.

### The Use of Tandem Repeats to Infer Phylogenetic Relationships Among Plant Genomes

While low-pass genome skimming of plant genomes is not adequate to analyze their gene space, repetitive sequences present in thousands of copies are sufficiently represented in this data. Repeat analysis using graph-based clustering methods allowed for computationally efficient and robust characterization of repetitive elements and provided much deeper insights into repeatome structure and evolution (Harkess et al., 2016; Doronina et al., 2017; McCann et al., 2020). Moreover, abundances of *de novo* identified repetitive elements were found to carry phylogenetic signals (Dodsworth et al., 2014, 2016, 2017). If assuming that repeat abundances are evolving through random genetic drift (Jurka et al., 2011), the abundances can be analyzed as continuous characters for phylogeny inference (Dodsworth et al., 2014). When using a genome proportion of 0.1% or higher, this method proved to be highly congruent with phylogenies inferred using other nuclear or plastome markers (Dodsworth et al., 2014, 2017; Bolsheva et al., 2019). Recently, Vitales et al. (2020) reported a novel approach of phylogenetic inference using repeats as markers. They utilized the RepeatExplorer2 similarity matrices and generated derived matrices which consist of the observed/expected read similarity values by considering the number of reads of each taxon that are represented in clusters. Thus, the matrices consist of pairwise sequence similarities, disregarding the number of reads for each species. By transforming these similarity matrices to distance matrices, they were able to build consensus networks for each dataset. Similar to the abundance-based method, the lineage-specific differences between homologous repeats are regarded to be regulated by random genetic drift in diversification, thus expected to carry phylogenetic signals (Jurka et al., 2011; Dodsworth et al., 2014; Vitales et al., 2020). However, it should be noted that tandem repeats undergo rapid turnover in plant (e.g., Koukalova et al., 2010; Renny-Byfield et al., 2013) and animal genomes (e.g., Sinha and Siggia, 2005; Cechova et al., 2019) and that their phylogenetic signals can be erased during long-term reproductive isolation and independent evolution of initially closely related genomes. As approaches using repeats as phylogenetic markers are still in their infancy, these phylogenetic inferences should be applied cautiously, along with other marker gene sets (Vitales et al., 2020), as done here for inferring phylogenetic relationships in Heliophileae.

## Data Availability Statement

The datasets presented in this study can be found in online repositories. The names of the repositories and accession numbers can be found in the Supplementary Tables 1–12.

## Author Contributions

ML and TM conceived the experiments. MD, MP, TM, PH, XG, PW, ZC, and IA-S conducted the study and/or processed the data. MD, XG, MP, TM, PW, IA-S, AV, KM, LM, and ML wrote the manuscript. All authors have read and approved the final manuscript.

## Conflict of Interest

The authors declare that the research was conducted in the absence of any commercial or financial relationships that could be construed as a potential conflict of interest.

## Footnotes

1
https://www.ncbi.nlm.nih.gov/Sequin

2
https://www.geneious.com

## Supplementary Material

Supplementary Table 1 List of *Heliophila* and *Chamira* accessions used in our study.

Supplementary Table 2 Chromosome numbers and genome sizes of *Heliophila* and *Chamira* species used for repeatome and plastome assembly.

Supplementary Table 3 Summary statistics of transcriptome assemblies.

Supplementary Table 4 List of species used in transcriptome analyses and phylogeny reconstruction based on transcriptome data.

Supplementary Table 5 List of selected chloroplast genomes and their respective GenBank accession numbers used in maternal phylogeny inference.

Supplementary Table 6 Repetitive elements investigated by FISH.

Supplementary Table 7 Inferred meso-WGD Ks peaks and estimated ages of WGD in the sampled *Heliophila* species and *C. circaeoides*.

Supplementary Table 8 Mixture modeling of synonymous divergence between *Heliophila* and *Chamira* species.

Supplementary Table 9 Summary of SNP frequency analysis based on transcriptome data.

Supplementary Table 10 Chloroplast genome assembly statistics of analyzed *Heliophila* and *Chamira* species.

Supplementary Table 11 Tandem repeats identified by RepeatExplorer2 and manual curation.

Supplementary Table 12 Pairwise sequence similarities between shared tandem repeats.

Supplementary Figure 1 BUSCO analysis of gene completeness.

Supplementary Figure 2 Fifty percent majority-rule consensus tree of the Bayesian inference of the ITS region.

Supplementary Figure 3 Time-calibrated plastome phylogeny of 59 Brassicaceae species.

Supplementary Figure 4 Dot-plot pairwise comparison of monomer consensus sequences of 16 identified tandem repeats shared among *Heliophila* species.

Supplementary Figure 5 Multiple and pairwise sequence alignments of 16 tandem repeats shared among 13 *Heliophila* species.

Supplementary Figure 6 Consensus network relationships of *Heliophila* species based on repeatome sequence similarities.

Supplementary Figure 7 Hierarchical clustering relationships of 15 *Heliophila* and *C. circaeoides* species produced from read abundance matrix produced by RepeatExplorer2 pipeline.
