# Corrigendum: Evolution of tandem repeats is mirroring post-polyploid cladogenesis in *Heliophila* (Brassicaceae)

**DOI:** 10.3389/fpls.2022.1054800

**Published:** 2022-10-25

**Authors:** Mert Dogan, Milan Pouch, Terezie Mandáková, Petra Hloušková, Xinyi Guo, Pieter Winter, Zuzana Chumová, Adriaan Van Niekerk, Klaus Mummenhoff, Ihsan A. Al-Shehbaz, Ladislav Mucina, Martin A. Lysak

**Affiliations:** ^1^ CEITEC, Masaryk University, Brno, Czechia; ^2^ NCBR, Faculty of Science, Masaryk University, Brno, Czechia; ^3^ Department of Experimental Biology, Faculty of Science, Masaryk University, Brno, Czechia; ^4^ South African National Biodiversity Institute (SANBI), Kirstenbosch, Cape Town, South Africa; ^5^ Institute of Botany, Czech Academy of Sciences, Prùhonice, Czechia; ^6^ Department of Geography & Environmental Studies, Stellenbosch University, Stellenbosch, South Africa; ^7^ Department of Biology, Botany, Osnabrück University, Osnabrück, Germany; ^8^ Missouri Botanical Garden, St. Louis, MO, United States; ^9^ Harry Butler Institute, Murdoch University, Perth, WA, Australia

**Keywords:** repetitive DNA, repeatome, whole-genome duplication (WGD), rDNA ITS, plastome phylogeny, Cruciferae, Cape flora, South Africa

**Error in Figure/Table**

In the published article, there was an error in **Figure 6** as published. The *in situ* localization of HeAre-*Gypsy* in *Heliophila arenaria* was erroneously duplicated and presented as localization of HeVar-*Chromo* in *H. variabilis*. The corrected Figure 6 and its caption appear below.

**Figure 6:**
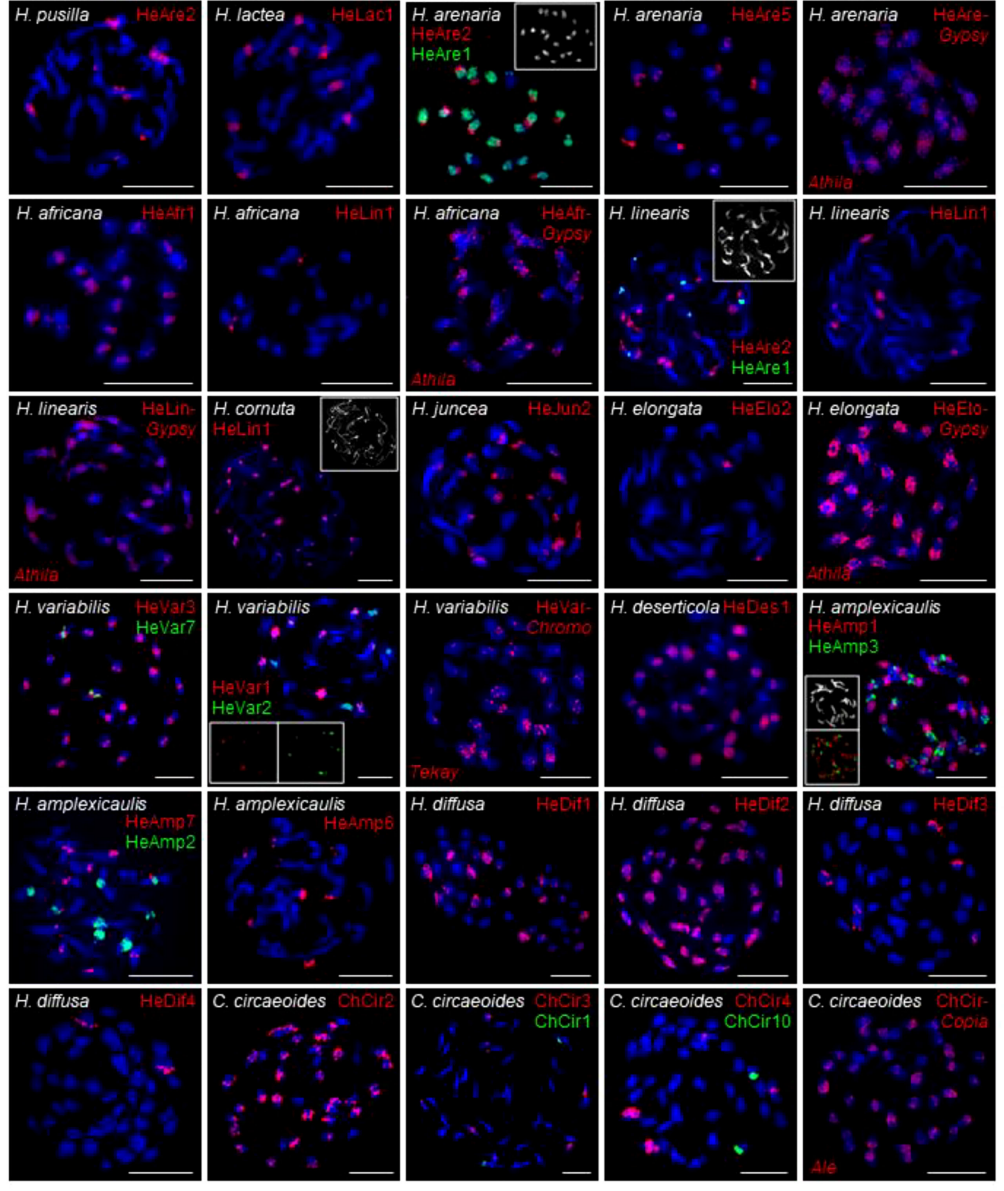
FISH localization of the selected tandem repeats and retroelements on mitotic metaphase chromosomes of *Heliophila* species and *C. circaeoides*. Chromosomes were counterstained by DAPI; FISH signals are shown in color as indicated. Detailed information on the localized repeats is provided in Supplementary Table 6. Scale bars, 10 μm.

The authors apologize for this error and state that this does not change the scientific conclusions of the article in any way. The original article has been updated.

## Publisher’s note

All claims expressed in this article are solely those of the authors and do not necessarily represent those of their affiliated organizations, or those of the publisher, the editors and the reviewers. Any product that may be evaluated in this article, or claim that may be made by its manufacturer, is not guaranteed or endorsed by the publisher.

