# Supplementary material for: Evolution of Tandem Repeats Is Mirroring Post-polyploid Cladogenesis in Heliophila (Brassicaceae)
Source: Front Plant Sci. 2021 Jan 12;11:607893. doi: 10.3389/fpls.2020.607893 (PMC7835680; doi:10.3389/fpls.2020.607893)

Supplementary Figure 1. BUSCO analysis of gene completeness.

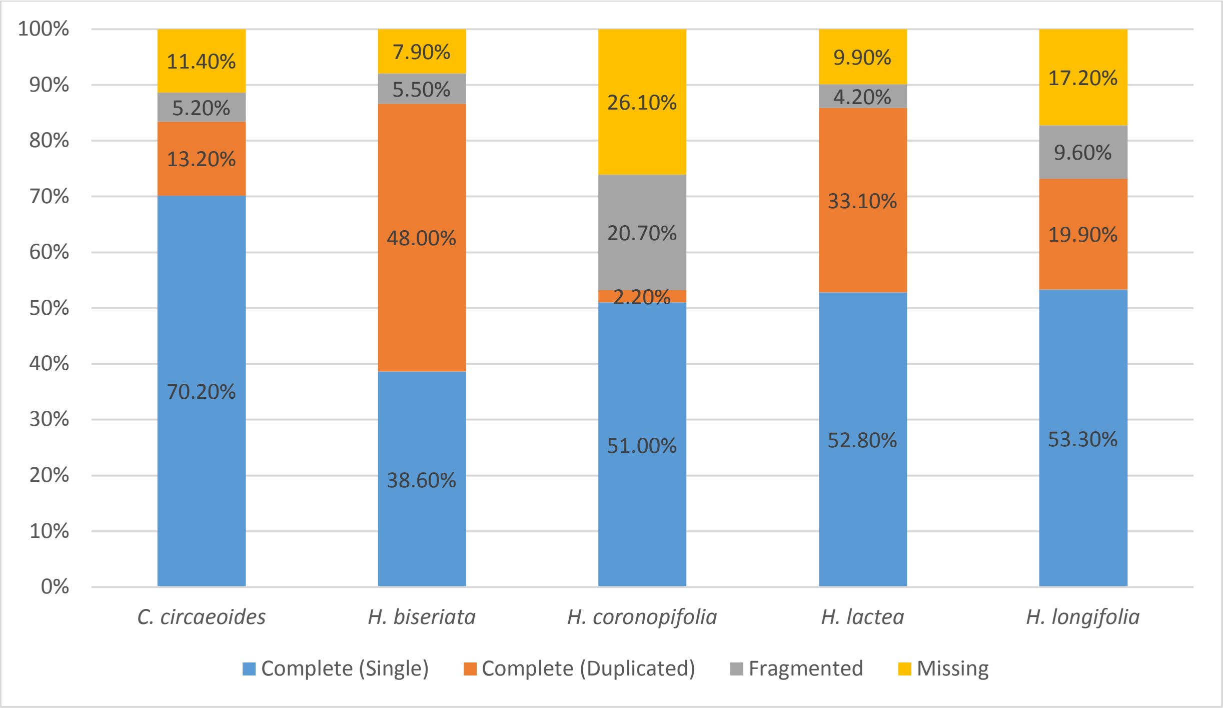

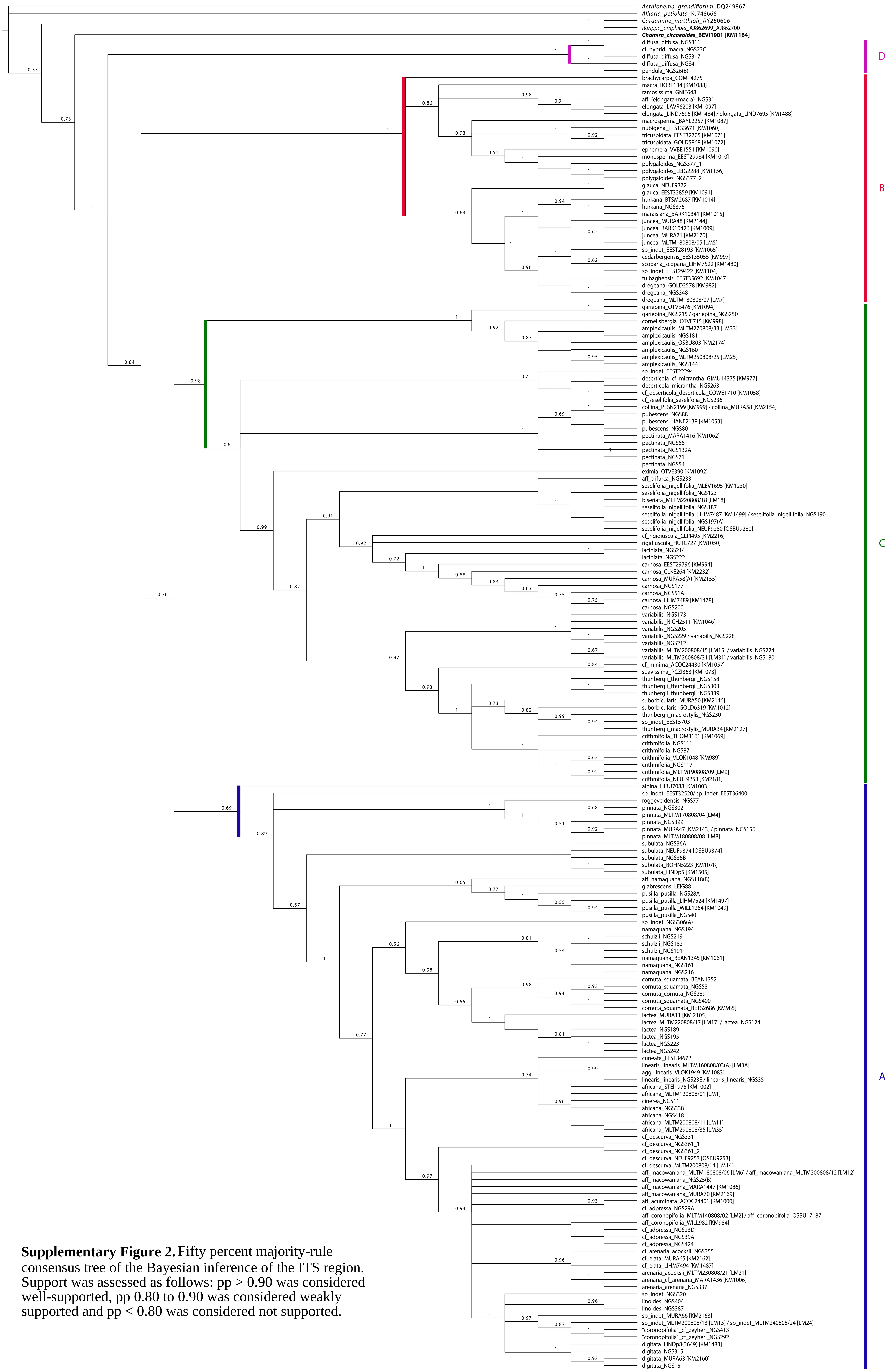

Supplementary Figure 2. Fifty percent majority-rule consensus tree of the Bayesian inference of the ITS region. Support was assessed as follows: pp > 0.90 was considered well-supported, pp 0.80 to 0.90 was considered weakly supported and pp < 0.80 was considered not supported.

Supplementary Figure 3. Time-calibrated plastome phylogeny of 59 Brassicaceae species. Branches of *Heliophila* species are colored according to their infraclades; Clade A: blue, Clade B: red, Clade C: green, Clade D: magenta. Divergence times are shown with 95% confidence intervals. Age constraints follow Hohmann et al. *(2015; Plant Cell 27: 2770-2784).*

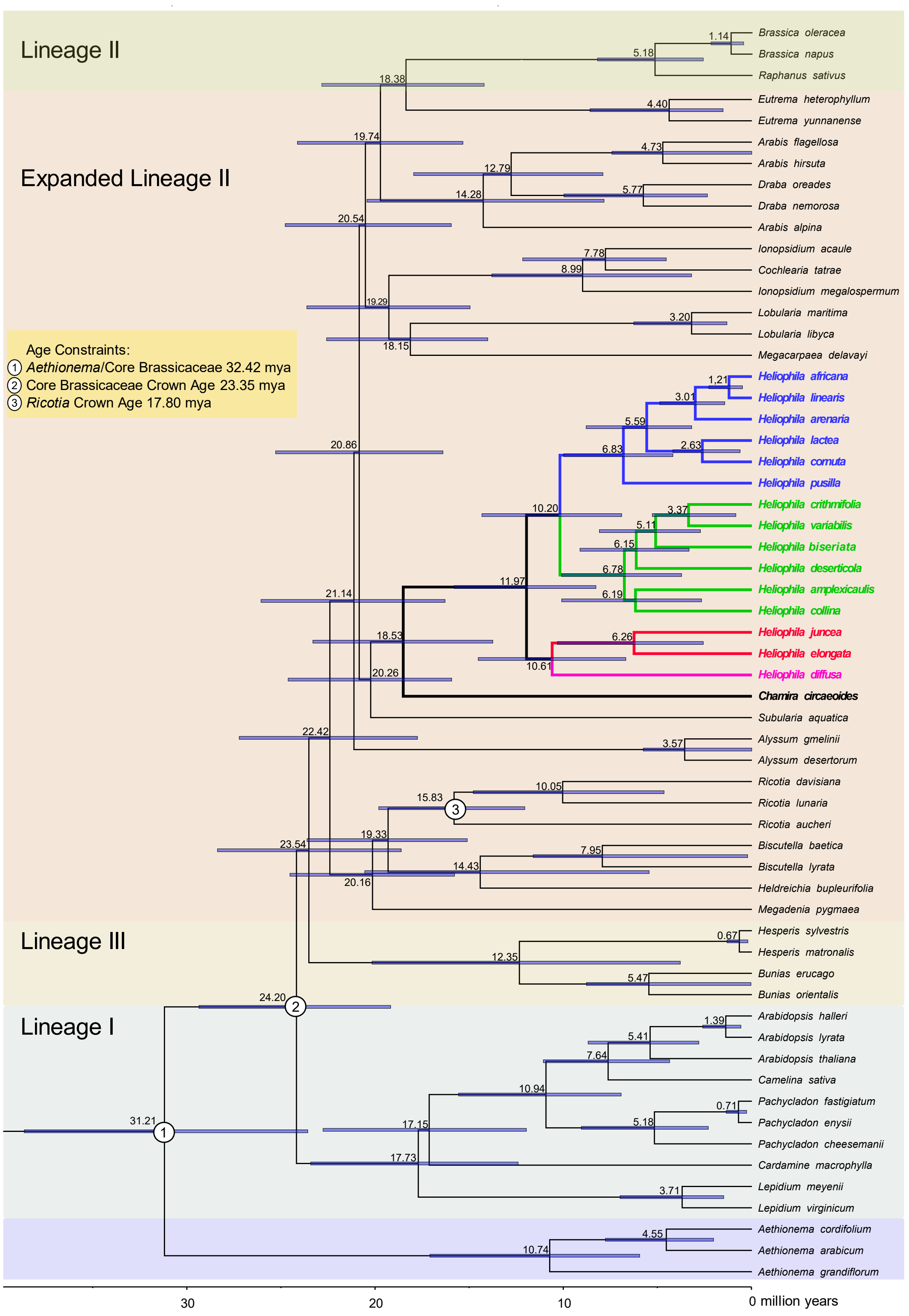

Supplementary Figure 4. Dot-plot pairwise comparison of monomer consensus sequences of 16 identified tandem repeats shared among *Heliophila* species. Tandem repeats are color-coded according to classification of *Heliophila* species to four infrageneric clades (Clade A: blue, Clade B: red, Clade C: green, Clade D: magenta). Supports Figure 4 and Supplementary Tables 11 and 12.

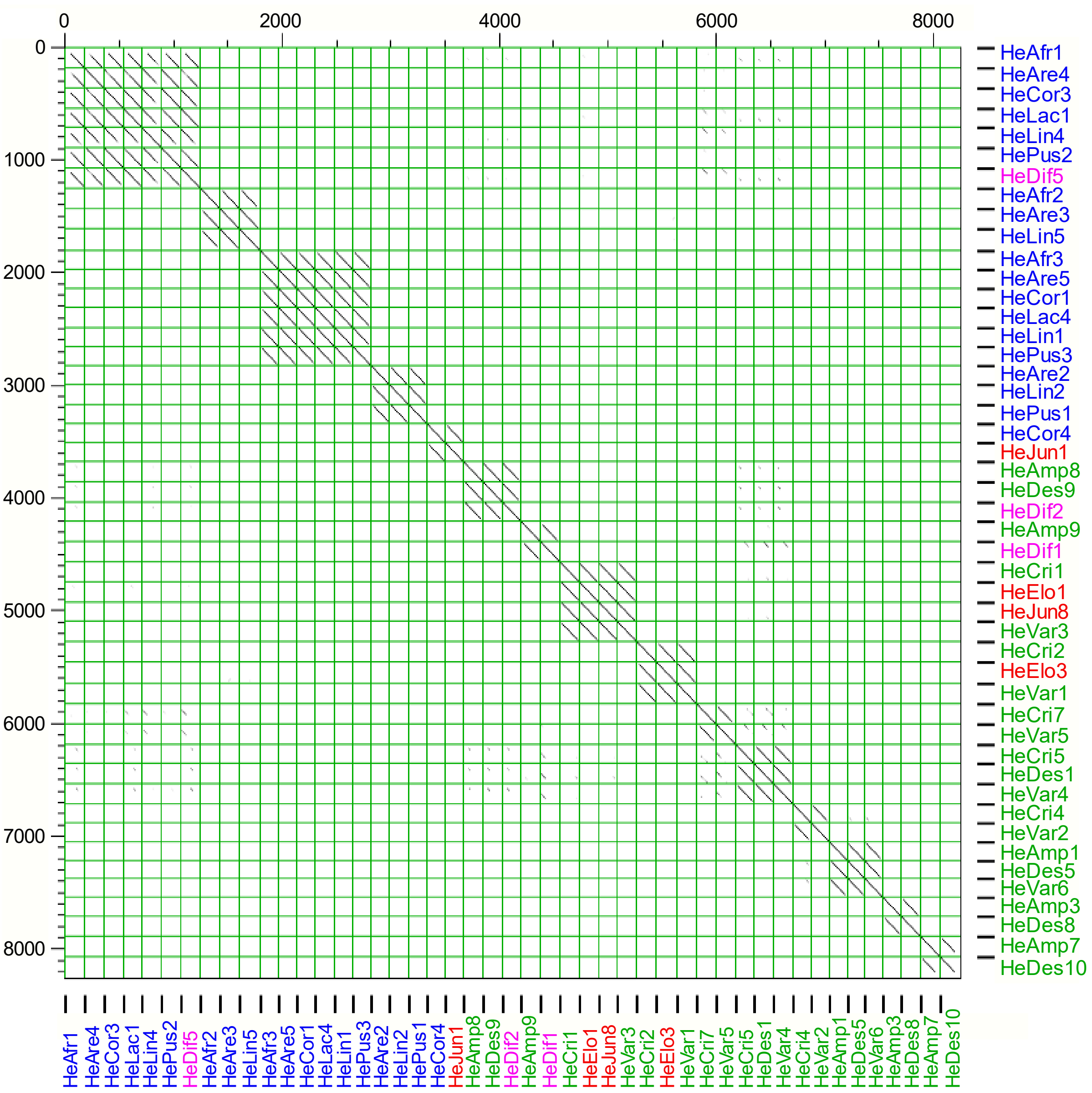

Supplementary Figure 5. Multiple and pairwise sequence alignments of 16 tandem repeats shared among 13 *Heliophila* species. Supports Figure 4 and Supplementary Tables 11 and 12.

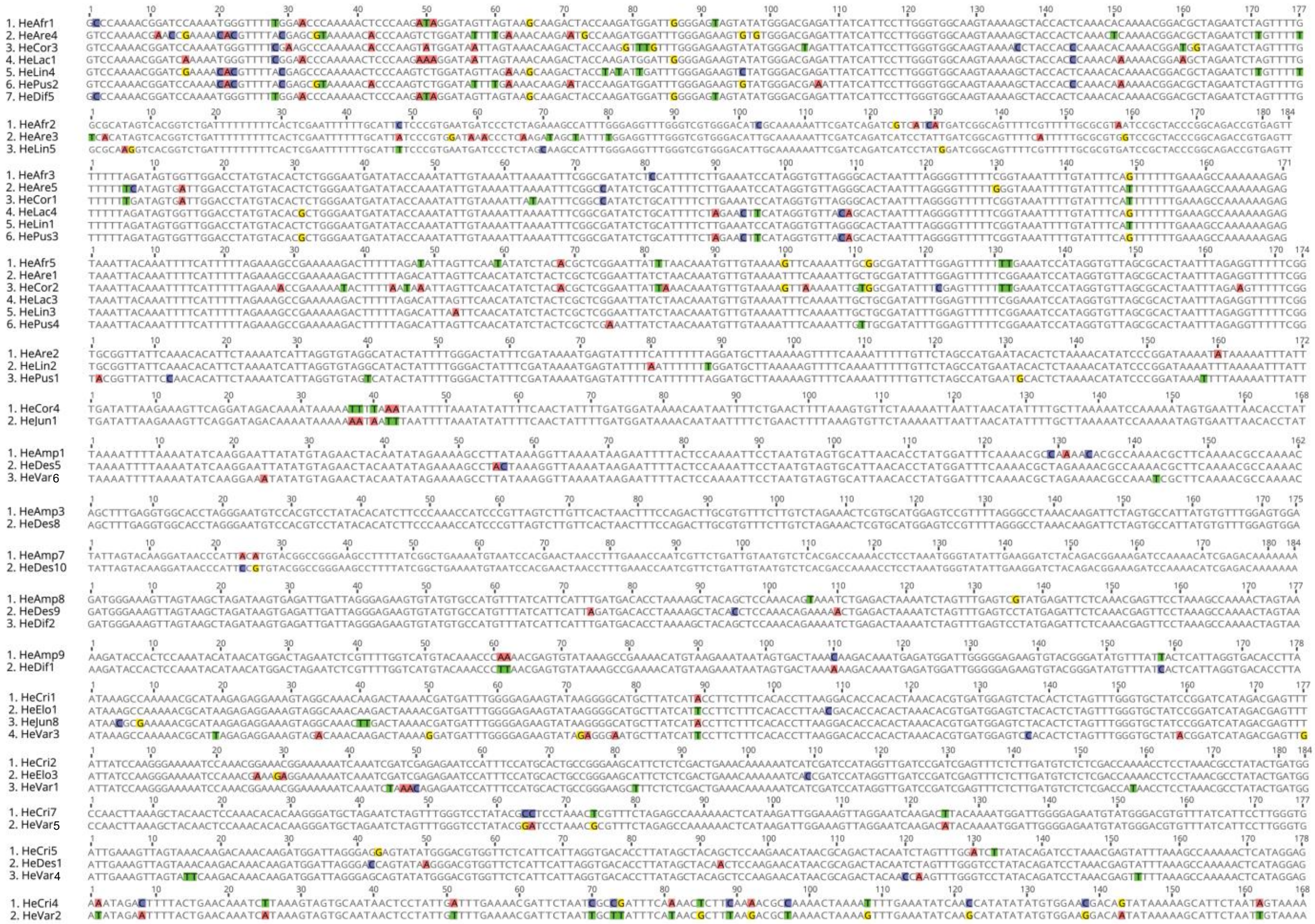

Supplementary Figure 6. Consensus network relationships of *Heliophila* species based on repeatome sequence similarities. (A) Clade C species were excluded, 39 similarity-based clusters were used to reconstruct the Neighbor-joining tree. (B) Clade A species were excluded, 29 similarity-based clusters were used to reconstruct the tree. Scale bars indicate mean branch length across all individual trees.

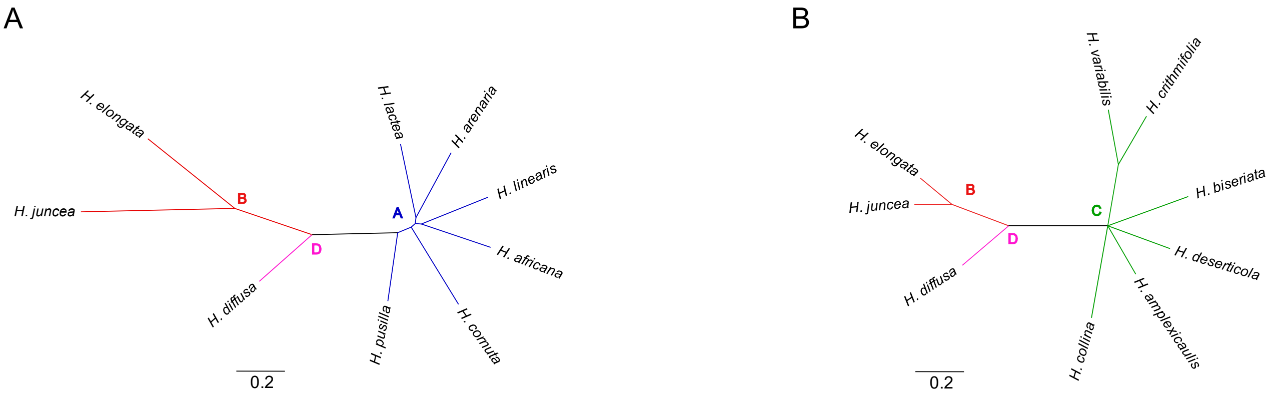

Supplementary Figure 7. Hierarchical clustering relationships of 15 *Heliophila* and *C. circaeoides* species produced from read abundance matrix produced by RepeatExplorer2 pipeline. A dendrogram which shows the clustering relationship of individual species was produced with hierarchical clustering algorithm of *pheatmap* R package. Rows represent individual clusters retrieved from RepeatExplorer2 comparative analysis and columns represent species. Colors indicate number of reads in the given cluster. Species are color-coded according to the four infrageneric clades in *Heliophila* (Clade A: blue, Clade B: red, Clade C: green, CladeD: magenta).

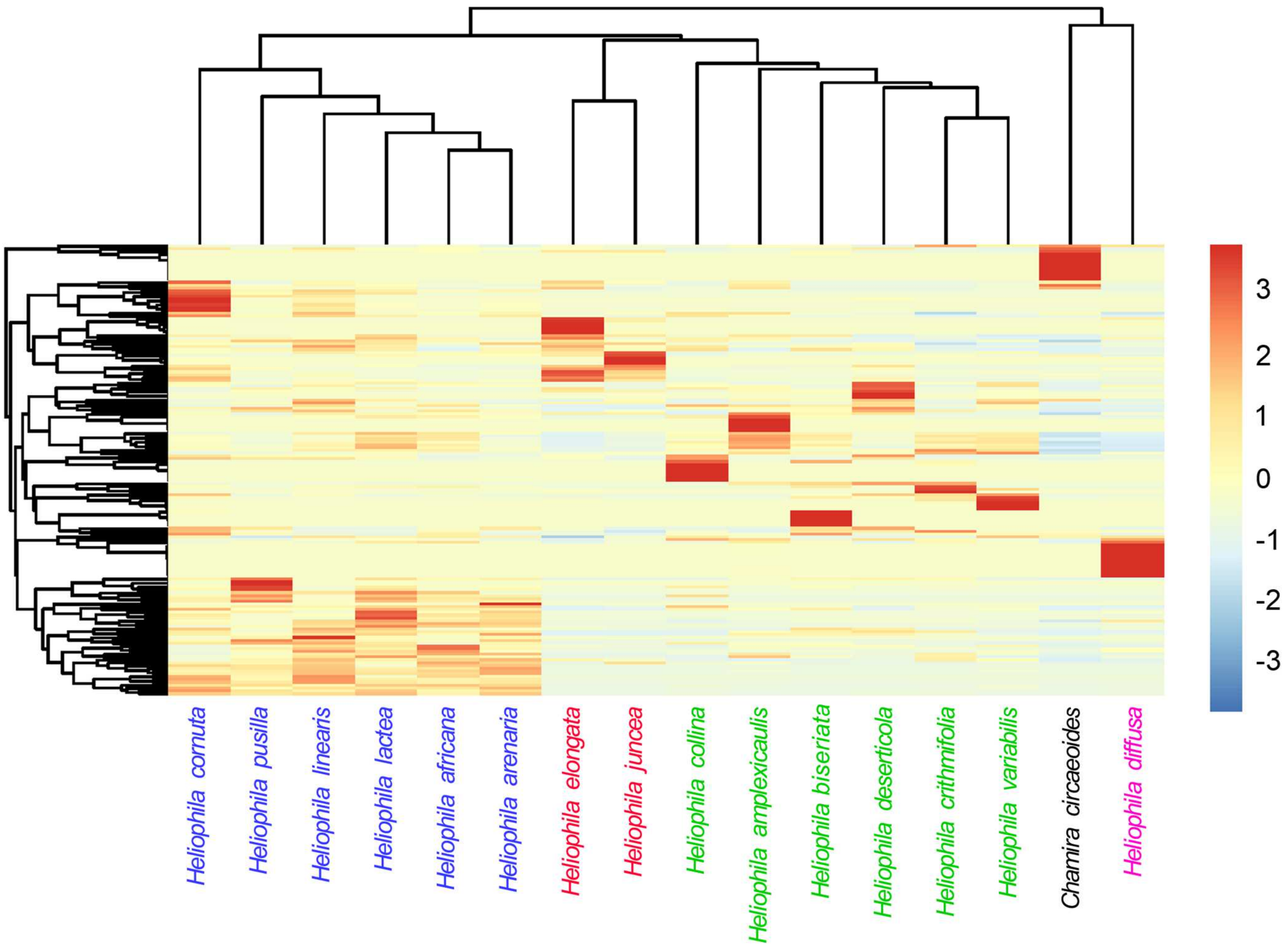

Supplement: Supplementary Figure 1 — BUSCO analysis of gene completeness. [file Data_Sheet_1.pdf]
